# Supplementary material for: Meta-analysis of neural systems underlying placebo analgesia from individual participant fMRI data
Source: Nat Commun. 2021 Mar 2;12:1391. doi: 10.1038/s41467-021-21179-3 (PMC7925520; doi:10.1038/s41467-021-21179-3)
Supplement: Supplementary file 1 — Supplementary Information [file 41467_2021_21179_MOESM1_ESM.pdf]

Meta-analysis of neural systems underlying placebo analgesia from individual participant fMRI data

Supplementary Information

Authors:
Matthias Zunhammer[1], Tamás Spisák[1], Tor D. Wager[2*], Ulrike Bingel[1*], The Placebo Imaging Consortium†

Affiliations:
[1] Center for Translational Neuro- and Behavioral Sciences, Dept. of Neurology, University Hospital Essen, Essen, Germany. [2] Cognitive and Affective Neuroscience Laboratory, Department of Psychological and Brain Sciences, Dartmouth College, Hanover, NH, USA.

*** Corresponding authors:** tor.d.wager@dartmouth.edu, ulrike.bingel@uk-essen.de

† Consortium authors: The Placebo Imaging Consortium
Lauren Atlas[3,4,5], Fabrizio Benedetti[6,7], Ulrike Bingel[1], Christian Büchel[8], Jae Chan Choi[9,10], Luana Colloca[11], Davide Duzzi[12], Falk Eippert[13], Dan-Mikael Ellingsen[14,15], Sigrid Elsenbruch[16], Stephan Geuter[17], Ted J. Kaptchuk[18], Simon S. Kessner[19], Irving Kirsch[18], Jian Kong[20], Claus Lamm[21], Siri Leknes[22,23], Fausta Lui[12], Alexa Müllner-Huber[21], Carlo A. Porro[12], Markus Rütgen[21], Lieven A. Schenk[8], Julia Schmid[24], Tamás Spisák[1], Nina Theysohn[25], Irene Tracey[26], Tor D. Wager[2], Nathalie Wrobel[27], Fadel Zeidan[28], Matthias Zunhammer[1]

[3]National Center for Complementary and Integrative Health, National Institutes of Health, Bethesda, Maryland, USA. [4]National Institute on Drug Abuse, National Institutes of Health, Baltimore, Maryland, USA. [5]National Institute of Mental Health, National Institutes of Health, Bethesda, Maryland, USA. [6]University of Turin, Turin, Italy. [7]Plateau Rosà Labs, Plateau Rosà, Switzerland. [8]Dept. of Systems Neuroscience, University Medical Center Hamburg-Eppendorf, Hamburg, Germany. [9]Yonsei University, Wonju College of Medicine, Wonju, South Korea. [10]Cham Brain Health Institute, Seoul, South Korea. [11]University of Maryland, Baltimore, Maryland, USA. [12]Dept. of Biomedical, Metabolic and Neural Sciences, University of Modena and Reggio Emilia, Modena, Italy. [13]Max Planck Institute for Human Cognitive and Brain Sciences, Leipzig, Germany. [14]Norwegian Center for Mental Disorders Research (NORMENT), Oslo University Hospital, Norway. [15]Dept. of Psychology, University of Oslo, Norway. [16]Dept. of Medical Psychology and Medical Sociology, Ruhr University Bochum, Bochum, Germany. [17]Johns Hopkins University, Baltimore, Maryland, USA. [18]Beth Israel Deaconess Medical, Harvard Medical School, Boston, Massachusetts, USA. [19]Dept. of Neurology, University Medical Center Hamburg-Eppendorf, Hamburg, Germany. [20]Massachusetts General Hospital, Harvard Medical School, Cambridge, Massachusetts, USA. [21]Social, Cognitive and Affective Neuroscience Unit, Dept. of Cognition, Emotion, and Methods in Psychology, Faculty of Psychology, University of Vienna, Vienna, Austria. [22]Dept. of Psychology, University of Oslo, Oslo, Norway. [23]Dept. Diagnostic Physics, Oslo University Hospital, Oslo, Norway. [24] Institute of Medical Psychology and Behavioral Immunobiology, University Hospital Essen, Essen, Germany. [25]Insitute for Diagnostic and Interventional Radiology and Neuroradiology, University Hospital Essen, Germany. [26]University of Oxford, Oxford, United Kingdom. [27]Karolinska Institute, Solna, Sweden. [28]Wake Forest School of Medicine, Winston-Salem, North Carolina.

Table of contents

Supplementary Methods and Results **4**

Study identification *4*
Criteria for study eligibility 4
Original study identification 4
Post-hoc study identification 4

Risk-of-bias-assessment *6*
Selection bias 6
Performance bias 6
Detection bias 6
Attrition bias 6
Study reporting bias 7
Other biases: unbalanced testing sequence in within-subject designs 7
Risk-of-bias summary 7
A note on external validity 7

Analysis details *8*
General 8
Brain coverage 8
Image alignment 8
Quality control of image signal 8
Smoothing 8
Presentation of pain vs baseline contrast 9
Meta-analysis 9
Labelling of outcome clusters 9
Responder analysis 9

Supplementary Figures **10**

Supplementary Figure 1: CONSORT flowchart of data-acquisition 10
Supplementary Figure 2: brain-coverage by number of subjects 11
Supplementary Figure 3: brain-coverage by study-level degrees of freedom 12
Supplementary Figure 4: placebo induced changes in pain-related activity (conservative sample) 13
Supplementary Figure 5: correlations of behavioral placebo analgesia and changes in pain-related brain activity (conservative sample) 14
Supplementary Figure 6: pain-related activity in experimental placebo imaging studies (non-placebo control condition only) 15
Supplementary Figure 7: between-study heterogeneity in pain-related activity 16
Supplementary Figure 8: effects of placebo-treatment on pain-related activity at peak voxels 17
Supplementary Figure 9: between-study heterogeneity versus placebo-treatment related effects 20
Supplementary Figure 10: exploratory comparison of placebo > control of studies using conditioning & suggestions with studies using suggestions only for placebo induction 21
Supplementary Figure 11: cerebral activity correlating with behavioral placebo analgesia at peak voxels 22
Supplementary Figure 12: between-study heterogeneity versus correlation of cerebral and behavioral placebo effects 28
Supplementary Figure 13: a comparison of placebo-related brain activation changes with regions contributing to the NPS. 29
Supplementary Figure 14: atlases used for similarity-based analysis of brain activity 30

Supplementary Tables **33**

Supplementary Table 1: study screening, eligibility checking, and retrieval 33
Supplementary Table 2: included studies: design, demographics, & heat stimulation 35
Supplementary Table 3: included studies: placebo conditions 39
Supplementary Table 4: included studies: functional neuro imaging acquisition characteristics 40
Supplementary Table 5: included studies: pre-processing and first-level analysis of neuroimages 41
Supplementary Table 6: experimental conditions selected for full and conservative analysis 42
Supplementary Table 7: risk of bias assessment according to the Cochrane risk-of-bias assessment tool: 43

Supplementary Table 9A: clusters of significant increase in pain-related activity — full sample, random effects analysis 45
Supplementary Table 9B: clusters of significant decrease in pain-related activity — full sample, random effects analysis 46
Supplementary Table 10: clusters of placebo-treatment induced reduction in pain-related activity — full sample, random effects analysis 47
Supplementary Table 11A: clusters of placebo-treatment induced increase in pain-related activity — full sample, fixed effects analysis 48
Supplementary Table 11B: clusters of placebo-treatment induced reductions in pain-related activity — full sample, fixed effects analysis 49
Supplementary Table 12: clusters showing a significant negative correlation between brain activity and behavioral placebo analgesia — full sample (sans between-subject studies), random effects analysis 50

Supplementary References **51**

Supplementary Methods and Results

Study identification

Study identification procedures have previously described in the Supplement of Zunhammer et al. (2018)[1] and are repeated below for convenience:

Criteria for study eligibility

Studies were defined eligible if…

a) …published in a peer-reviewed journal in English language
b) …based on an original investigation
c) …including human participants
d) …obtaining functional neuroimaging data of the brain during evoked pain
e) … involving pain delivered under stimulus intensity-matched placebo and control conditions, where "Placebo treatment" was defined as any condition where the experimental context suggested that an effective analgesic treatment was applied, including verbal suggestions and conditioning procedures that reinforced participants' expectations of reduced pain, following the categorization of placebo paradigms introduced in Ref.[2]. Accordingly, non-placebo control conditions that involved no treatment, ineffective treatment, hidden (in contrast to open) treatment, and unconditioned (in contrast to conditioned) treatment were considered eligible.

Original study identification

Studies were identified through the following sources:

a) an initial online-search of the electronic bibliographic database MEDLINE via PubMed on May 21st 2015 using the search term:
((placebo effect[Title/Abstract]) OR placebo analgesia[Title/Abstract]) AND fMRI OR PET.
b) by enriching initial search results with studies identified in an earlier meta-analysis of author TW[2,3]. Search results in these preceding peak-voxel-based meta-analyses were obtained by "identified using literature searches in PubMed and Google Scholar, the authors' personal libraries, and examining references of relevant papers."
c) through recommendations by collaborating investigators.

Studies identified are listed in Supplementary Table 1, the data-acquisition process is illustrated in Supplementary Figure 1. Authors MZ, UB, and TW screened the titles and abstracts of all records retrieved; studies that provisionally met eligibility criteria were assessed for eligibility by examining the full text. Study eligibility was determined in a joint discussion of authors MZ, UB, and TW. Agreement between reviewers was accomplished in a joint discussion. There were no studies where the decision for inclusion/exclusion was a matter of ambiguity (see Supplementary Table 1).

Post-hoc study identification

An exploratory post-hoc literature search was performed on March 10th 2018 to account for the fact that considerable time had passed between the initial study search and the completion of the meta-analysis. We searched pubmed and Thomson Reuters Web of Science from the beginning of 2015 to the present day using the following (extended) search terms:

- Pubmed:
(placebo effect OR "placebo analgesia" OR "placebo effect"[MeSH]) AND ("functional magnetic resonance imaging" OR fMRI OR PET OR "functional neuroimaging" OR ASL OR fMRI[MeSH] OR "functional neuroimaging"[MeSH]) AND (pain OR pain[MeSH] OR analgesia OR noci) NOT (Review[Filter] OR Editorial[Filter] OR Comment[Filter])*
- Web of Science (WoS, searching: all databases):
TS=("placebo effect" OR "placebo analgesia") AND TS=(pain OR analgesia OR noci) AND TS=("functional magnetic resonance imaging" OR fMRI OR POET OR "functional neuroimaging" OR ASL) Refined by: [excluding] DOCUMENT TYPES: (REVIEW OR EDITORIAL OR CASE REPORT)*

After removing duplicates, author MZ screened titles abstracts and assessed full-texts for eligibility. The post-hoc analysis indicated that at least six eligible studies[4–9] (with a total *N* of 196) were published after the initial study search in 2015 and therefore missed by the present meta-analysis (Supplementary Table 1).

Data collection

Investigators of all eligible studies were contacted and invited to share data. Specifically, we requested participant-level summary images (statistical parameter estimates, or beta-images) representing any relevant experimental condition. The decision to collect pre-processed, summarized participant-level images (aka 1st-level images) was based on the following considerations:

1. Raw images may contain personal information (meta-data, anatomical features captured in images) that could make individual research-participants identifiable. Sharing of such images across workgroups may only be possible after consultation of local ethics committees. Additional measures (removal of meta-data and face-masking) would have to be taken to ensure participant anonymity. Meta-data of statistical summary images from SPM and fsl do not contain individual information by default and therefore safeguard anonymity.
2. The analysis of neuroimaging data is an elaborate multi-step process that involves numerous analysis decisions. A multitude of opinions exist regarding the optimal analysis pipeline, especially when it comes to expressing an experimental (stimulus) protocol as a statistical model (most often a GLM). The "optimal" analysis depends on many considerations, some of which cannot be based on data alone. We relied on the expertise of the original researchers to choose the best approach for the data at hand.
3. When collecting raw imaging data, the associated experimental stimulus protocols have to be collected for analysis. These often do not come in a standardized format. Re-modelling the statistical analysis in terms of pain and placebo-conditions is therefore laborious and error prone. Further, re-modelling the data requires many decisions on the side of the meta-analyst that cannot be pre-registered. This poses a potential source of "researcher degrees of freedom" and therefore bias that we wanted to avoid.

Risk-of-bias-assessment

Risk-of-bias identification procedures were re-applied analogue to Ref.[1] (Supplement), with the difference that we assessed the risk of bias regarding voxel-wise whole-brain activity. Note that most risks of bias apply to both meta-analyses, regardless of the target outcome, therefore the assessment below largely is a replication of our earlier assessment; conclusions in risk of bias were largely identical in respect to performance bias, detection bias, and study reporting bias.
Author MZ evaluated each study with respect to selection bias, performance bias, attrition bias, detection bias, report bias, and biases introduced by the use of within-subject designs (sequence effects) using to the Cochrane risk of bias tool[10]. All judgments were based on single-subject data, information taken from the published manuscripts, or personal communication with the study authors, following this order of priority.

Selection bias
Non-random sampling and group allocation of research participants can be a considerable source of bias. While, the issue is of major importance in between group designs, requirements are relaxed in within subject designs, as all participants undergo both treatments[11]. Most studies in our sample followed a within subject design and were therefore judged “low risk of selection bias” (Supplementary Table 3). In summary, selection bias due to non-random allocation of participants to placebo/control conditions was judged as low in most studies (Supplementary Table 3).

Performance bias
Awareness of the allocated experimental condition by participants and study personnel is considered the major source of performance bias in clinical trials[10]. However, the issue of blinding in experimental placebo research is controversial: The knowledge of being treated is considered constitutional for the placebo phenomenon[12]. Further, the treatment provider and her behaviour are seen as major factors driving the placebo effect [13]. Placebo studies with blinded study participants or treatment providers[12] may underestimate the placebo effects typical for clinical settings. On the other hand, the fact that full blinding is conceptually difficult in experimental placebo studies does not imply that performance bias is not a problem[12]. The lack of blinding in placebo studies makes it difficult to discern “true” placebo effects, i.e. perceived and actual symptom improvements, from “false” placebo effects, i.e. apparent improvements due to demand characteristics / altered reporting behaviour[12]. Thus, so-called “demand characteristics” (participant’s tendency to report what they believe they *should* report, independent of experience) and other biases in judgement and decision making can influence behavioural placebo effects, which is a major reason to also examine physiological outcomes.
No studies in our sample blinded participants or experimenters, with the exception of one between-group study that blinded subjects in respect to group allocation[14]. Therefore, we concluded high risk of performance bias for the present meta-analysis, as voxel-wise brain activity related to demand characteristics cannot be discerned from brain activity related to placebo analgesia with certainty.

Detection bias
It is a common problem in neuroimaging research that image pre-processing pipelines and statistical analysis involve numerous analysis choices. These do not only tempt analysts to cherry-pick favourable results, but also state a multiple comparison problem[15]. Blinding of analysts to the nature of experimental conditions and pre-specification of analysis parameters could exclude this type of bias.
No included study reported analyst blinding (Supplementary Table 3). Moreover, the pre-processing pipelines and 1st-level models of imaging analyses varied considerably (Supplementary Table 5). Since our meta-analysis relies on the original first-level analyses, choices by the original analysts may affect results of the present meta-analysis. Analysis pipelines may have been chosen so as to favour some brain regions over others. We therefore judged the risk for detection bias as high.

Attrition bias
Study drop-out and exclusion of participants may systematically affect study outcomes, especially when one experimental condition is affected more than another, or when participants are selected based on outcomes. Supplementary Table 7 provides a general overview on the amount of missing imaging data in respect to different experimental stages of the original studies. For one study[16] insufficient information was available to determine these figures. For the remaining studies, we found that our meta-analysis included 84% of participants included in the original studies, 95% of participants successfully completing fMRI testing, and 99% of subjects included in the original analysis. Main reasons for the discrepancy between participants tested and participants completing measurements were problems with neuroimaging and pain stimulation equipment, which are unlikely to affect our outcome systematically. Main reasons for the discrepancy between participants completing measurements and participants analysed in the original studies were exclusions due to imaging artefacts and due to excessive head movements, which are also unlikely to affect placebo effects systematically. Data from 6 out of 16 subjects for one[17] and 2 out of 19 subjects for another study[18] were unavailable doe to failure of data-storage. Given the relatively low attrition rate and the fact that most studies are within-subject studies, where

missing participants affect all experimental conditions alike, we conclude that attrition bias is unlikely to affect the outcomes of our meta-analysis.

Study reporting bias
The underreporting of studies with non-significant (“negative”) results is a prevailing problem in biomedical research[19] that has been suggested to affect experimental placebo research[12]. Underreporting of studies with non-significant behavioural placebo effects may inflate the effect sizes of our current meta analysis, by biasing the study sample towards placebo responders. Further, imaging studies yielding no activation clusters or clusters in unorthodox regions may have been underreported, although we are not aware of such a case. Based on these results we conclude that there the risk of report bias was unknown for the present analysis.
Of note, the present study is based on single-subject whole-brain summary images, as obtained in the original analyses. The non-reporting of peak activations is therefore not a problem and consequently the risk of reporting bias of the present analysis is lower than in previous peak-based meta-analysis approaches (e.g. Ref.[2]).

Other biases: unbalanced testing sequence in within-subject designs
Sequence effects (e.g. habituation or sensitization) may confound treatment-effects in within-subject designs when the order of experimental conditions is not balanced or randomized. An overview on the sequence of treatment conditions in within-subject studies is provided in Supplementary Table 3. Single-subject data on the sequence of conditions was available for all but three studies, two studies reported balanced testing[20], only for one study no information about testing sequence was available[21]. Several studies tested placebo and control conditions in an alternating fashion, reducing the risk of sequence confound[16,17,22–24]. Two studies tested placebo and control conditions in a fixed pre-placebo (control) vs. post-placebo sequence[18,25]. These studies were excluded from conservative analysis. All remaining studies had balanced designs in respect to the sequence of placebo and control. Overall, sample imbalance for studies was low: placebo conditions were tested after control conditions in 54% of participants. Based on these figures we judged the overall risk of bias due to unbalanced sequence of testing as low.

Risk-of-bias summary
In summary, we concluded high risk of bias for voxel-wise brain activity. Main reason for this decision was the unresolved issues of distinguishing real placebo analgesia from report bias and the risk that detection bias due to non-blinding of analysts affected results.

A note on external validity
The Cochrane risk-of bias tool focusses on the assessment of internal study validity. Beyond this tool, we identified an issue of external validity, that may affect the conclusions of the present meta-analysis. Two studies[20,26] (accounting for 20.7% of the total sample, see Supplementary Table 3) pre-selected placebo-responders. This practice constitutes no bias in terms of internal validity and merely limits the generalizability of results. Mixing studies with and without responder-selection in a meta-analysis may entail an over-representation of placebo responders and therefore inflate our effect size estimates.

Analysis details

General

The present analysis was not pre-registered, yet performed corresponding to the analysis plan for Zunhammer et al. (2018)[5] (see https://osf.io/n9mb3/), with the difference that single-voxel brain responses were the main outcome, not NPS-responses. Of note, statistical thresholds, were not pre-defined in the original analysis plan. Therefore we provide maps for several established thresholding methods, i.e. uncorrected at $p < .001$ (parametric p-values), family-wise error (FWER) corrected at $p < .05$ (non-parametric permutation-based p-values), with and without probabilistic threshold-free cluster enhancement[6]).

All analyses were performed with MATLAB (v 2016b). All images were re-sliced to a voxel size of 2*2*2 mm using *SPM 12*'s imgcalc function before further analysis. The meta-analysis was based on the algorithms used in Cochrane's *RevMan 5*[28], implement as custom MATLAB functions. The functions and the complete analysis are available at: www.github.com/mzunhammer/PlaceboMetaAnalysis.

Brain coverage

Binary signal/no-signal masks were created for each subject. The resulting voxel-coverage maps were summarized within and across studies to determine the available sample size/missing data at each brain voxel). Brain-voxels which represented less than four participants were excluded at study-level. Subsequently brain-voxels missing in > 10% of participants (total sample) were excluded from further analysis to keep the sample-size comparable across the brain. The decision to exclude such voxels was not pre-established before analysis. The coverage map for the full sample are shown in Supplementary Figure 2. The study-level coverage after excluding missing voxels is shown in Supplementary Figure 3

Image alignment

We checked alignment to Montreal Neurological Institute (MNI)-space and image coverage by visually comparing binary signal/no-signal masks and study summaries for pain > baseline against the standard MNI template supplied with SPM (avg152T1.nii). All studies showed satisfactory alignment with the template upon visual inspection, with no single-participant outliers.

Quality control of image signal

Correct data labelling was ascertained in correspondence with the original authors. Outlier screening for excessive random error in imaging signal was guided by the assumption that imaging and statistical artefacts should mainly affect the absolute and relative signal intensities of grey matter, white matter, csf, and extracerebral signal. Raw and absolute parameter estimates for each tissue were obtained by calculating the dot-product of each individual image with SPM8's tissue probability maps grey.nii, white.nii, csf.nii and (inverted) brainmask.nii. Mahalanobis distance and scatterplots were then used to identify suspect cases on a within-study basis. Further, the design matrices (SPM.mat, design.mat) used for first level analysis in the original analyses were evaluated for irregularities, if available.

Outlier screening identified 63 cases showing unusual absolute and/or relative activity in white matter, grey matter, CSF, or extra-cerebral space. These suspect images underwent further evaluation using histograms and visual examination. In total, 12 subjects were confirmed as outliers, showing radio-frequency-, magneticsusceptibility-, or spike-like-artifacts (6), extreme values (4), or evidence for errors in the original design matrices (SPM files) (2). Outliers were retained in full, but excluded from the conservative analysis.

Smoothing

The statistical summary images collected differed in terms of image smoothness (see Supplementary Table 5). Between-study Differences in smoothing kernel may impact negatively on the comparability of single studies and the statistical weight of individual studies to the meta-analysis. However, no measures were taken to equalize image smoothness before meta-analysis based on the following considerations:

- The main purpose of equalizing image smoothness is to achieve a better comparability of studies.[8] However, the present study primarily aimed at was to summarize brain activity across studies, not to make comparisons between individual studies.
- "One disadvantage of post hoc smoothness equalization is that it requires that all scanners be smoothed to that of the most smooth scanner in the set"[8]. Equalizing smoothing would entail a loss in statistical power and mapping-accuracy.

Presentation of pain vs baseline contrast
For the pain vs baseline comparison we pooled placebo and control conditions based on four considerations:

1. For some studies[29] only pooled estimates of the main effect of pain were available, the map based on "control images only" would not show the complete sample.
2. The pooled map that is optimal for comparing the pain and the placebo contrasts, as the two contrasts are orthogonal[30]. Comparisons based on the baseline-contrast, only would be bias comparisons, as it would reflect peculiarities of the control condition.
3. Pooling reduces within-subject variance and therefore robustness of results
4. The range of effect sizes observed for the pain vs baseline comparison was about 7 times greater than that observed for the placebo vs control comparison, so placebo-related effects do not affect the visualization of the pain vs baseline comparison at large.

Meta-analysis
For outcome comparisons within studies we used Hedges' *g*, which is the (mean difference / standard deviation (SD))*J, where J is a correction factor for small sample bias ($J = 1 - 3/(4*df - 1)$) [10]. For within-subject studies Hedges' g_{rm} was used, which is defined as: mean within-subject difference / SD_{diff} * sqrt(2.*(1-*r*))**J*, where SD_{diff} is the SD of within-subject differences and *r* is the correlation between repeated measures [31,32]. For three studies (Supplementary Figure 6), imaging data were only available as separate contrasts for pain activation and placebo conditions. For these studies no within-subject correlation *r* of pain-related activity under placebo- and control- conditions could be computed. For these studies Hedges' g_{rm} was obtained by imputing the mean within-subject correlation observed across all other within-subject studies. Treatment effects and correlations between cerebral treatment effects and ratings were summarized across studies using the generic inverse-variance (GIV) weighting method with DerSimonian and Laird random effects [28,32] Fisher's *Z*-transformation was applied before and after summarizing correlations [32]. Significance thresholds ($\alpha < .05$) and *p*-values correct for multiple comparison at family wise error level (p_{FWER}), were obtained by performing a non-parametric, Monte-Carlo (2000 re-samples) permutation-test based on the maximum *z*-score, corresponding to the maximum-*t* approach described by Nichols and Holmes (2002)[33]. To determine significance thresholds ($\alpha < .05$) and *p*-values corrected multiple comparison for the between-study heterogeneity estimates, the same permutation approach was applied to the maximum-*Q* (χ^2) statistic [10].

Labelling of outcome clusters
The fsl (version 5.0.10) function "cluster", as implemented in the atlasquery automation script (autoaq), was used to label thresholded summary images, automatically. The Harvard Cortical and Subcorical Atlases[34], the Oxford Thalamic Connectivity Atlas[35], the Probabilistic Cerebellar Atlas[36], and the Talairach Daemon (TD) Atlas [37] were used in this order of preference (as provided in fsl 5.0.10). Labels with a probability < .1 were omitted. White matter labels were omitted for brevity, except when no non-white matter label with a probability > .1 was available (low tissue probability implies white-matter).

Responder analysis
We initially planned another analysis including only participants showing an above-median behavioural placebo response for each study ("responder analysis", see https://osf.io/n9mb3/). However, we've replaced this analysis with the correlation analysis of behavioural and cerebral placebo responses, as the dichotomization of continuous outcomes is suboptimal in terms of statistical power and can yield misleading results[38].

Supplementary Figures

Supplementary Figure 1: CONSORT flowchart of data-acquisition

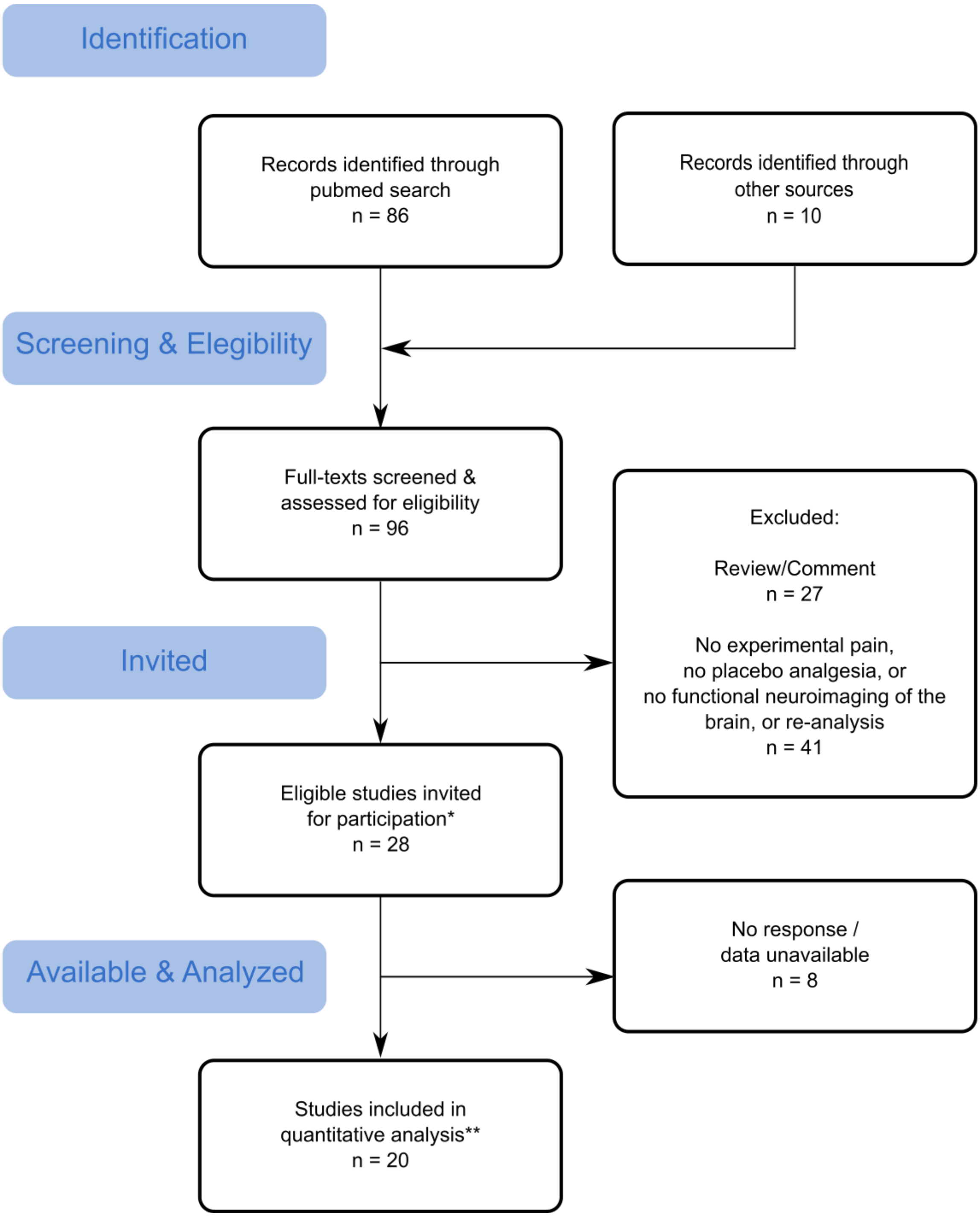

* IPD for all eligible studies were sought. ** All available studies were analyzed.

Supplementary Figure 2: brain-coverage by number of subjects

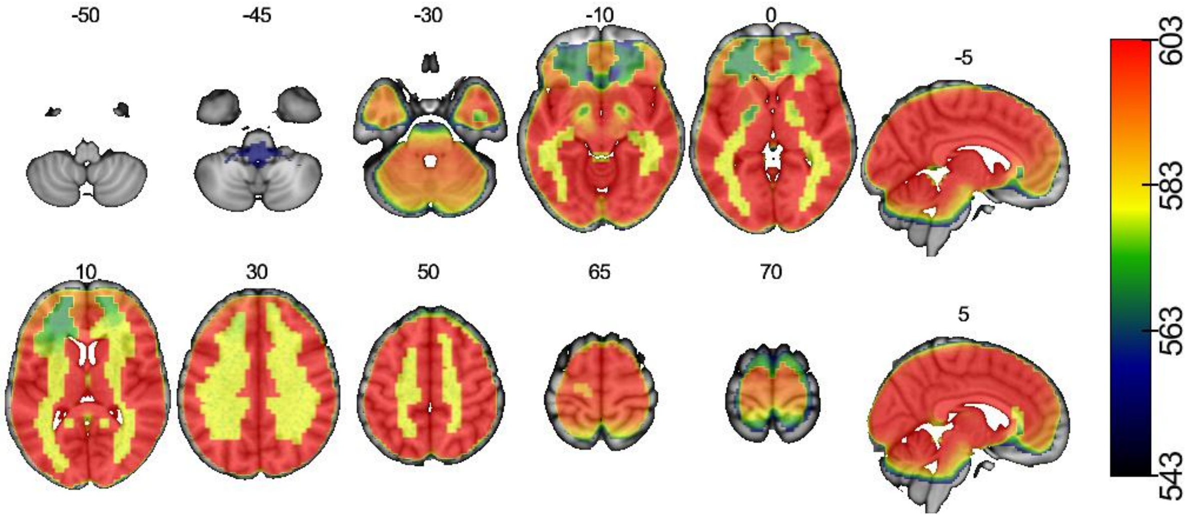

Number of participants with non-missing data (full sample), voxel-wise, projected onto the MNI152 brain template. Areas with more than 10% missing subjects were excluded from further analysis. The full sample analysis was based on 191118 brain-voxels (2*2*2 mm). n = 543 to 603 individuals from 17 to 20 independent studies per voxel. Source data (results as 3d-volumes) are provided at *https://osf.io/n9mb3/*.

Supplementary Figure 3: brain-coverage by study-level degrees of freedom

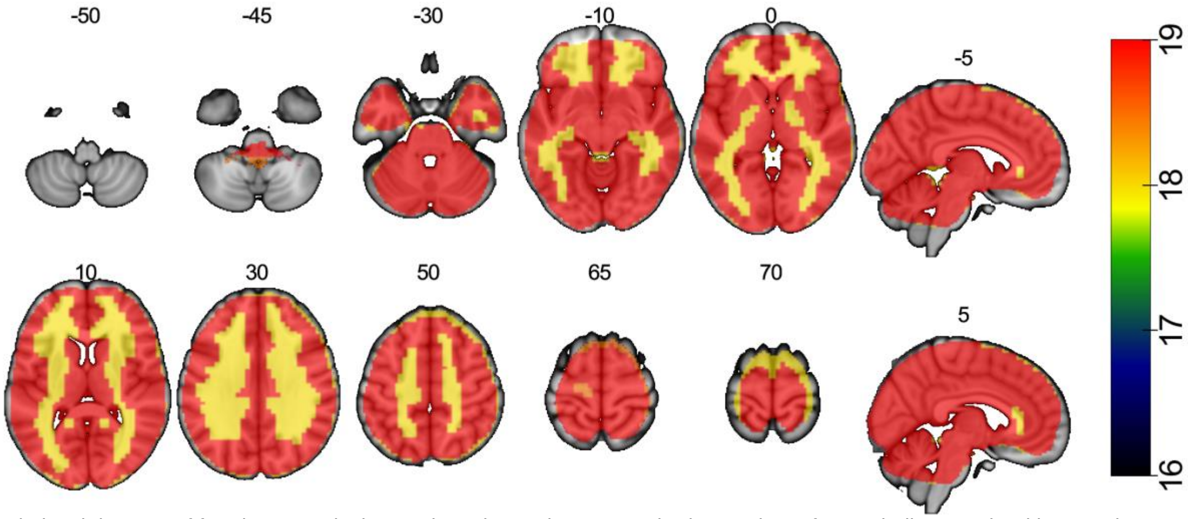

Study-level degrees of freedom, voxel-wise, projected onto the MNI152 brain template after excluding voxels with more than 10% missing subjects (see Supplementary Figure 1). The majority of included voxels (78%) represented results for all 20 studies (df = 19, red). The remaining voxels represent 19 studies (22%, df = 18, yellow) and a small minority of voxels 18 or 17 studies (0.1%, df = 17 or 16, red). Scale: df: [16; 19]; n = 543 to 603 individuals from 17 to 20 independent studies per voxel. Source data (results as 3d-volumes) are provided at https://osf.io/n9mb3/.

Supplementary Figure 4: placebo induced changes in pain-related activity (conservative sample)

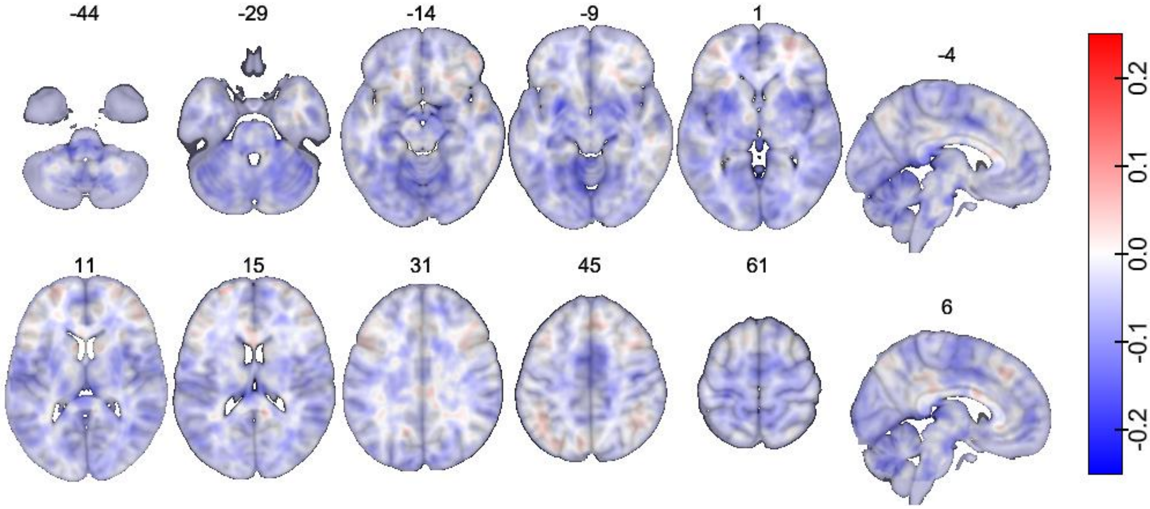

Standardized effect size *g* for the contrast $pain_{placebo}$ $pain_{control}$. Sagittal sections cut the hemisphere proximal to the viewer. Range *g*: [-0.23, 0.18]; n = 373 to 414 individuals from 13 to 16 independent studies per voxel. Un-thresholded effect sizes (Hedges' *g*). Red denotes increased, blue denotes decreased pain-related activity under placebo, compared to control conditions. Only a single voxel in the cerebellum (x = 50, y = –54, z = –30, Crus I 80%), showed a statistically significant (g = -0.19 ± 0.05 (SEM), τ^2 = 0, n = 381, z-score = –3.84, p_{FWER} = .041) de-activation. Activity increases did not reach statistical significance. Source data (results as 3d-volumes) are provided at https://osf.io/n9mb3/.

Supplementary Figure 5: correlations of behavioral placebo analgesia and changes in pain-related brain activity (conservative sample)

A

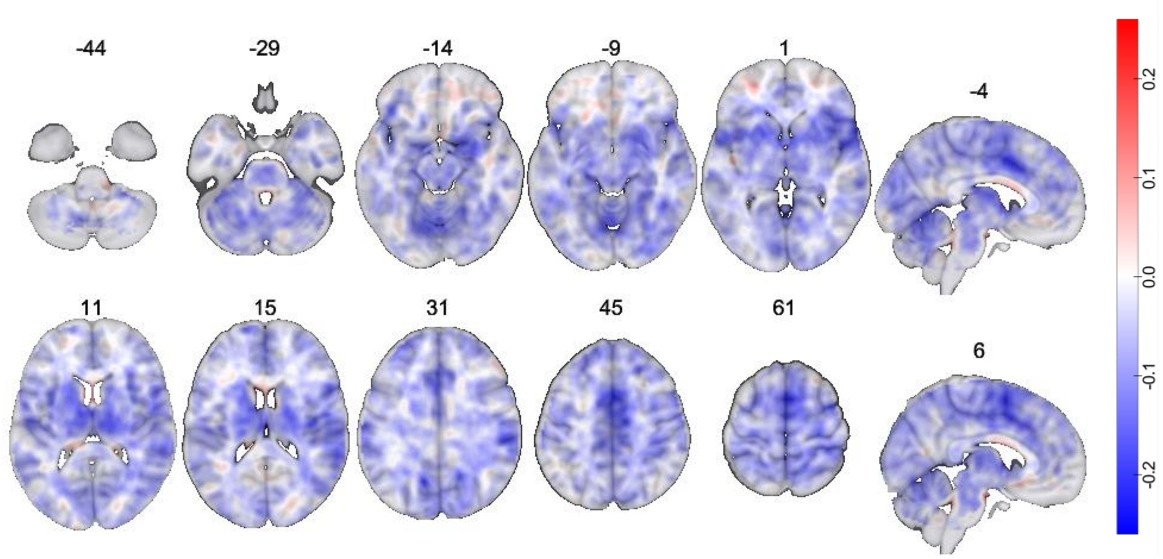

B

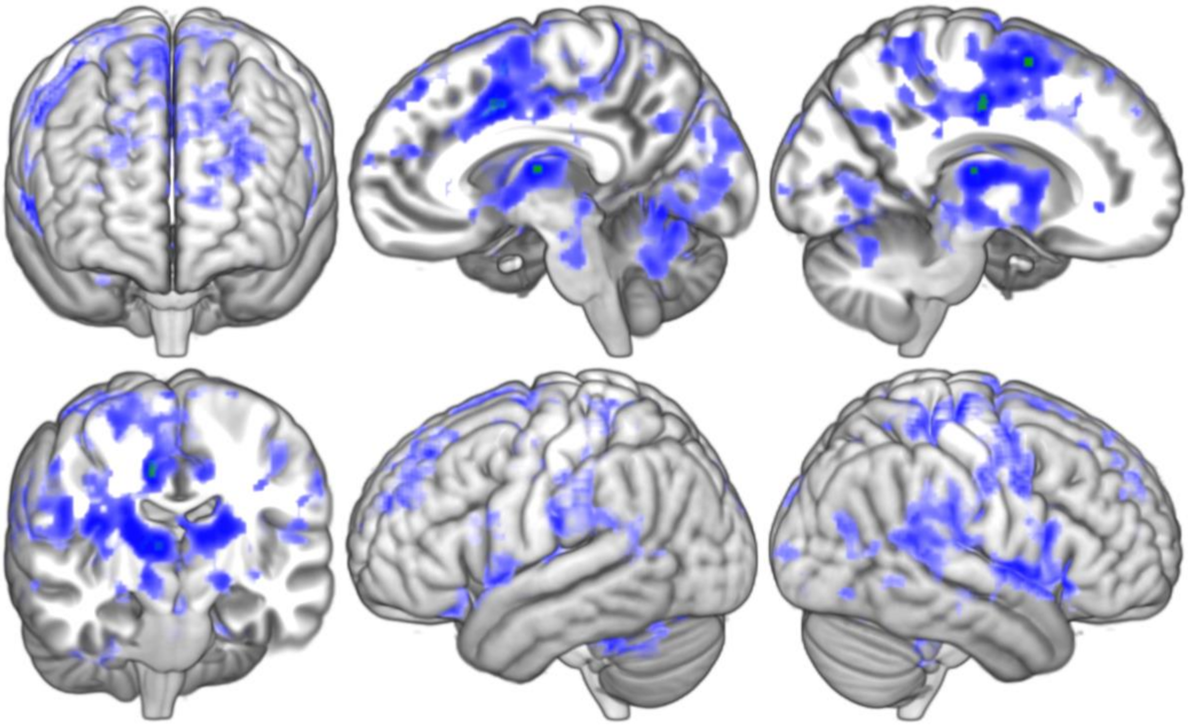

By-subject correlation between behavioral placebo analgesia ($pain_{control}$ $pain_{placebo}$) and placebo-related activity changes ($pain_{placebo}$ $pain_{control}$). Conservative sample excluding between-group studies (individual estimates of behavioral placebo analgesia not possible), high risk-of-bias studies and outlier subjects. Sagittal sections cut the brain hemispheres proximal to the viewer.

Panel A: un-thresholded Pearson's *r*. Red denotes positive (i.e. increased activity associated with larger placebo analgesia), blue denotes negative correlations (i.e. decreased activity associated with larger placebo analgesia). Range: r = [-0.27; 0.14]. Scale: *r* = [–0.28, 0.28], n = 373 to 414 individuals from 13 to 16 independent studies per voxel. Source data (results as 3d-volumes) are provided at https://osf.io/n9mb3/.

Panel B: statistically significant (two-sided $p < .05$, FWER corrected permutation test) negative correlations at voxel (green) and cluster level (blue, pTFCE-enhanced) according to a random (study-)effects analysis. Positive correlations did not reach statistical significance. Source data (results as 3d-volumes) are provided at https://osf.io/n9mb3/.

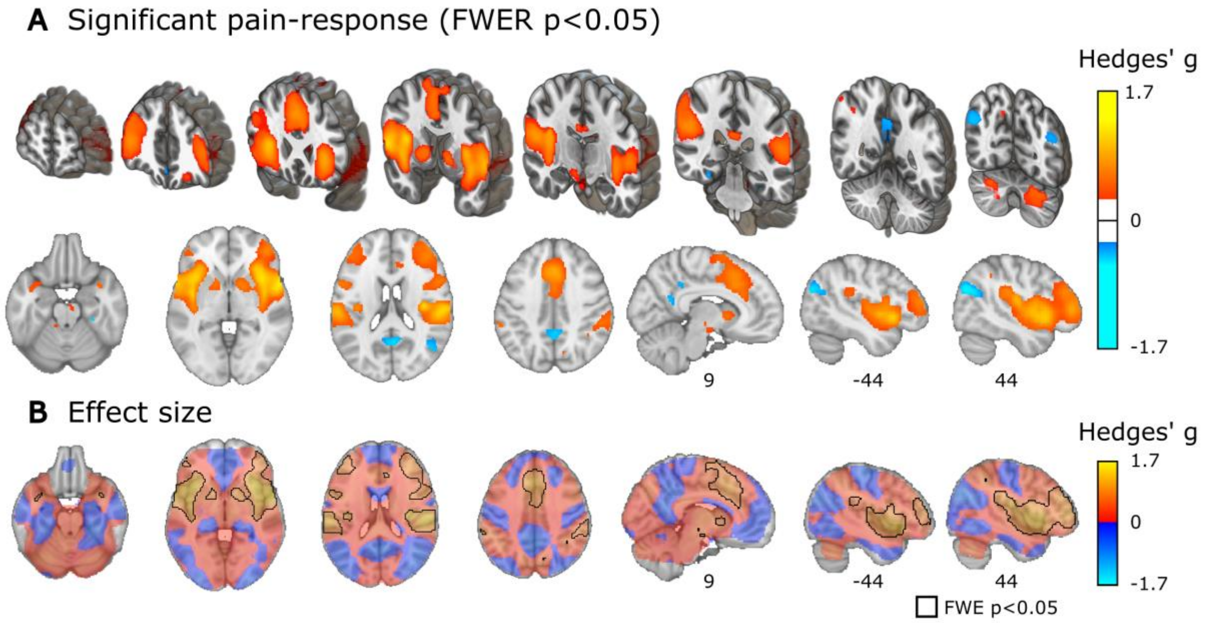

Supplementary Figure 6: pain-related activity in experimental placebo imaging studies (non-placebo control condition only)

A Statistically significant pain-responses (permutation test, controlled for FWER, two-sided p < 0.05); **B** whole-brain unthresholded standardized effect size *g* of acute pain stimulation > baseline, non-placebo control conditions only (FWER-corrected permutation test results are delineated as a back contour); range *g*: [-0.82, 1.68]; n = 434 to 494 individuals from 15 to 18 independent studies per voxel (for two studies, pain > baseline conditions were only available as pooled contrast). Three dimensional coronal slices are equidistantly distributed from *y* = 60 to -68 mm. Axial slices range equidistantly from *z* = -22 to 42 mm. Custom coordinates for sagittal slices are displayed in mm. Source data (results as 3d-volumes) are provided at https://osf.io/n9mb3/.

Supplementary Figure 7: between-study heterogeneity in pain-related activity

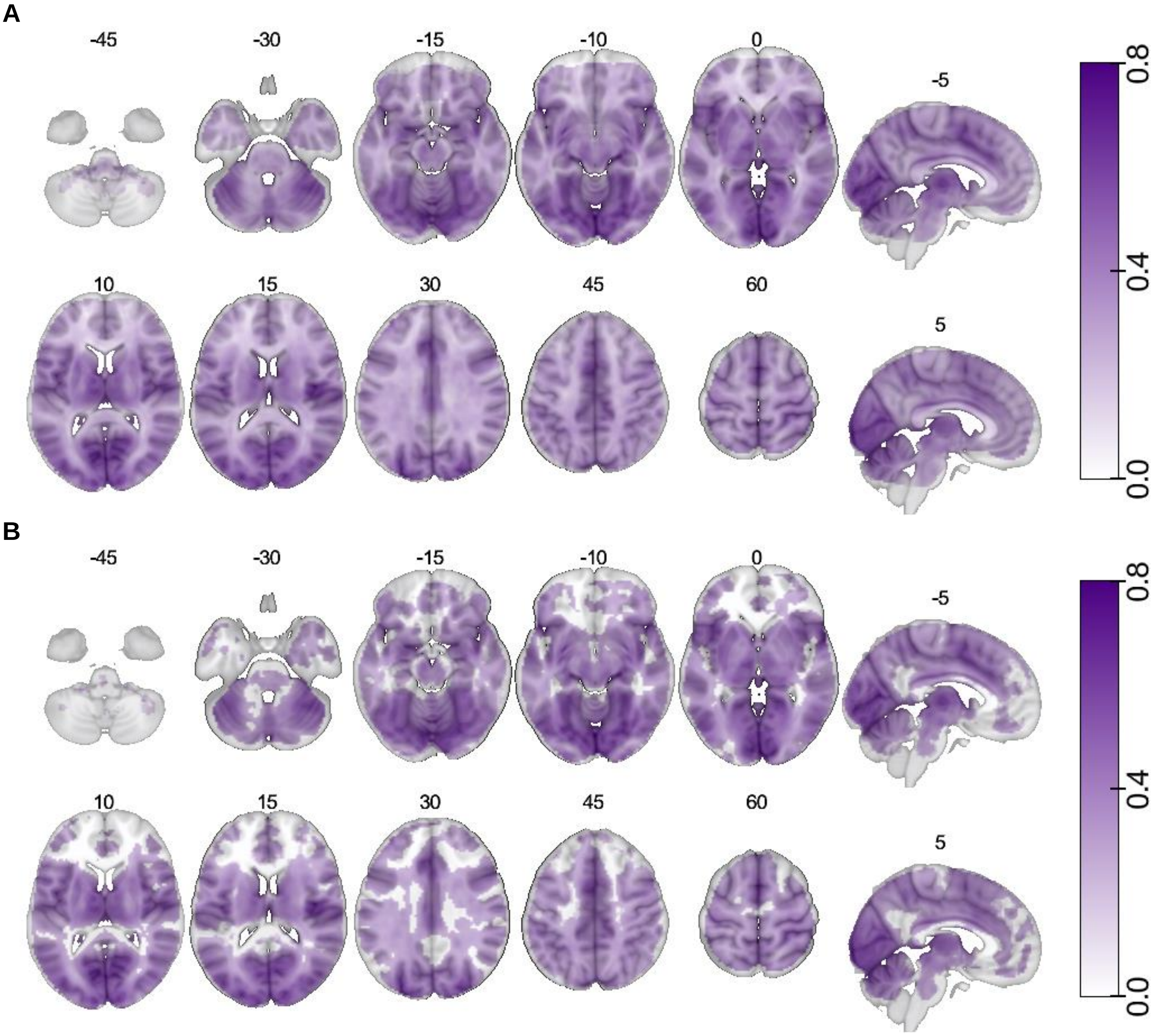

A unthresholded τ-values (estimated SD of effect size *g* due to between-study heterogeneity, scale τ: [0; 0.8]. range τ: [0, 1.07]) for the contrast pain stimulation > baseline (pooled across placebo and control conditions). **B** regions of statistically significant between study-heterogeneity (permutation test, controlled for FWER, one-sided $p < .05$). Scale: n = 543 to 603 individuals from 17 to 20 independent studies per voxel. Source data (results as 3d-volumes) are provided at https://osf.io/n9mb3/.

Supplementary Figure 8: effects of placebo-treatment on pain-related activity at peak voxels

A Insula

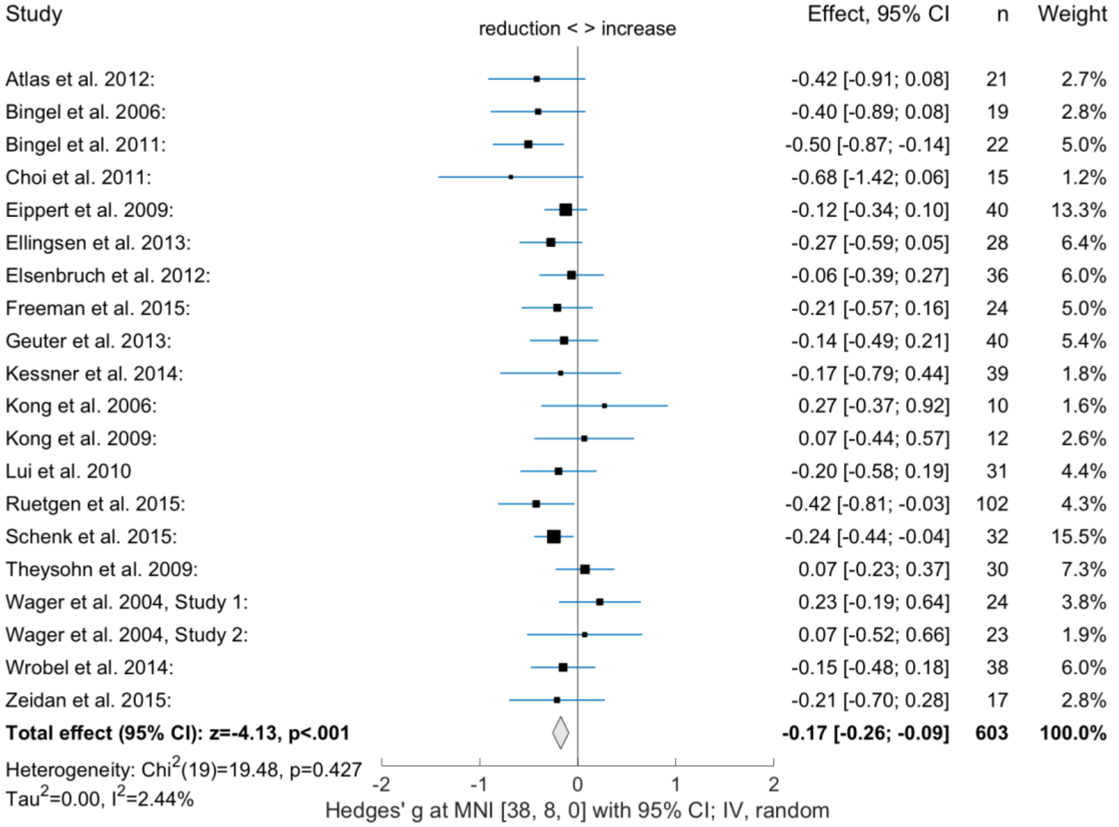

Source data (results as 3d-volumes) are provided at https://osf.io/n9mb3/.

B habenula / corpus callosum near splenium

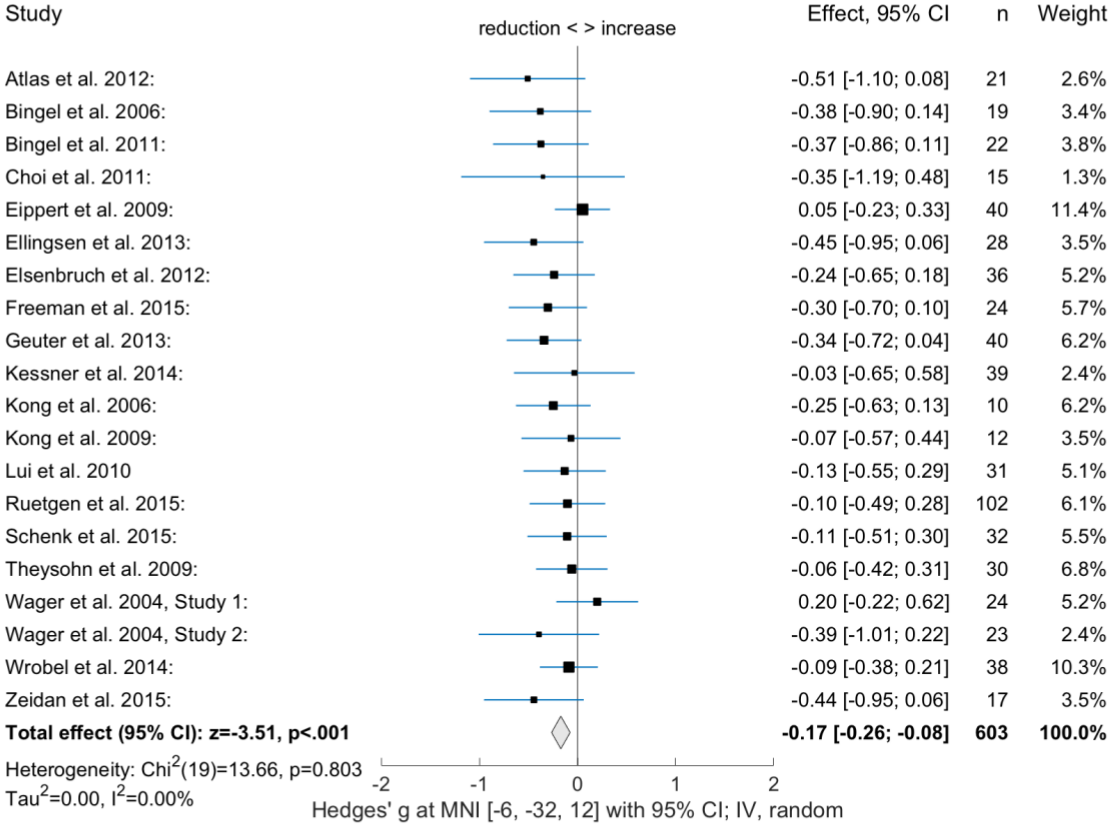

Source data (results as 3d-volumes) are provided at https://osf.io/n9mb3/.

C cerebellum (crus I)

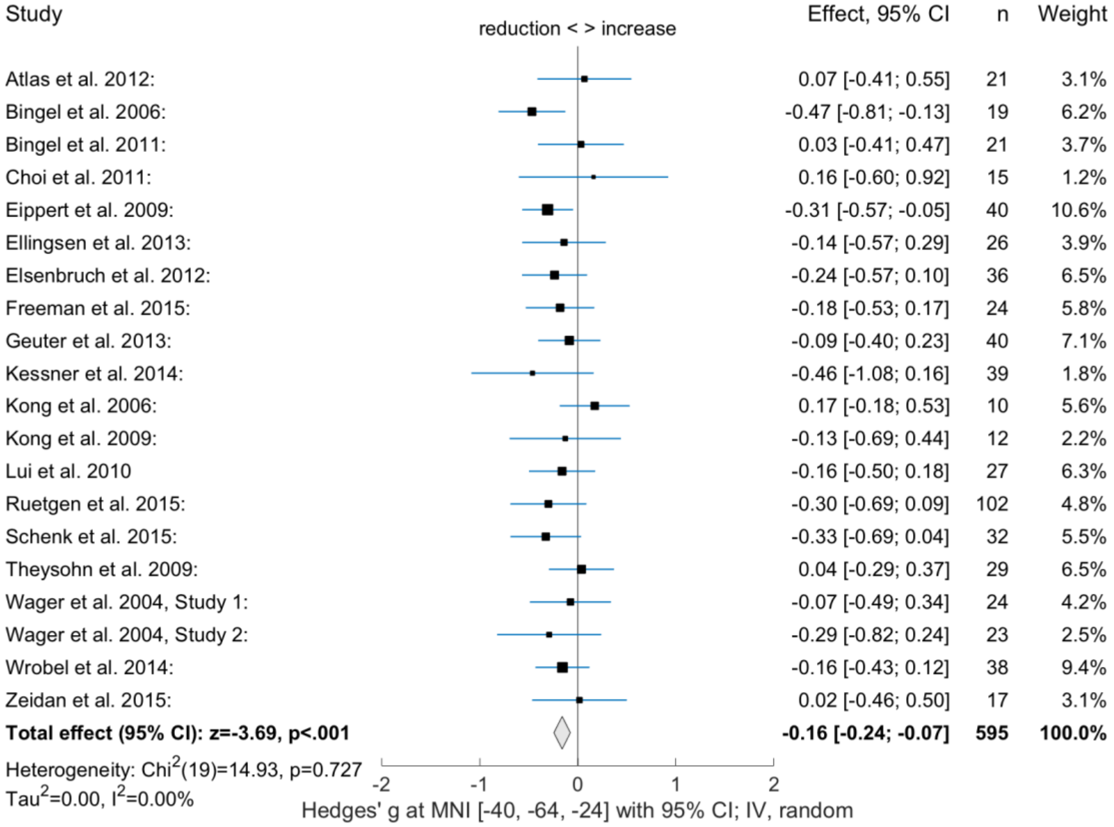

Source data (results as 3d-volumes) are provided at https://osf.io/n9mb3/.

Supplementary Figure 9: between-study heterogeneity versus placebo-treatment related effects

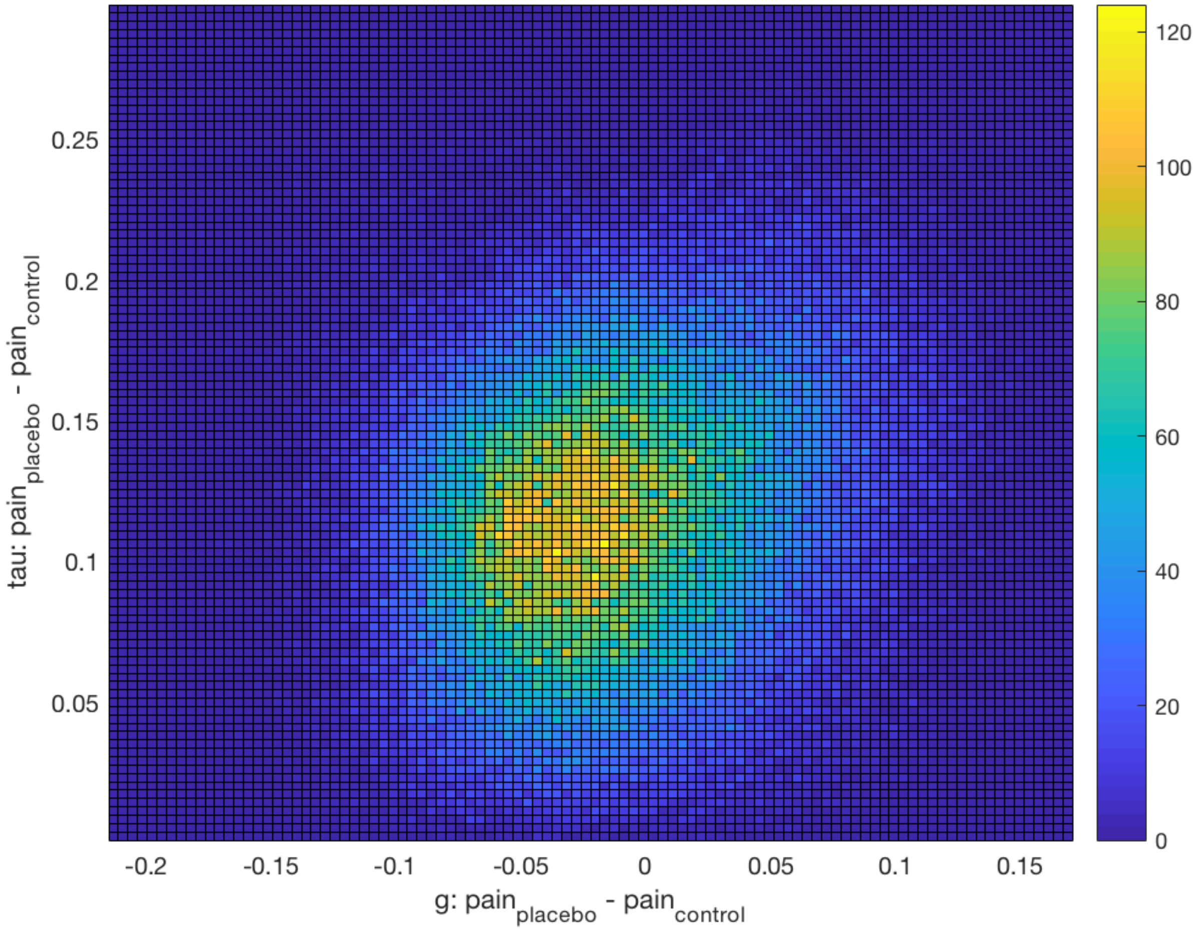

Across all brain-voxels (n = 191118 voxels, full sample), there was a small, positive, statistically significant correlation (r = .191, 95% CI [.187, .196], $p < .001$) between effects of placebo treatment and between-study heterogeneity estimate τ. Voxels where $\tau = 0$ (25% of voxels) and $\tau > .3$ (10 voxels) were excluded from the plot (but not the correlation analysis) for illustration purposes. Source data (results as 3d-volumes) are provided at https://osf.io/n9mb3/.

Supplementary Figure 10: exploratory comparison of placebo > control of studies using conditioning & suggestions with studies using suggestions only for placebo induction

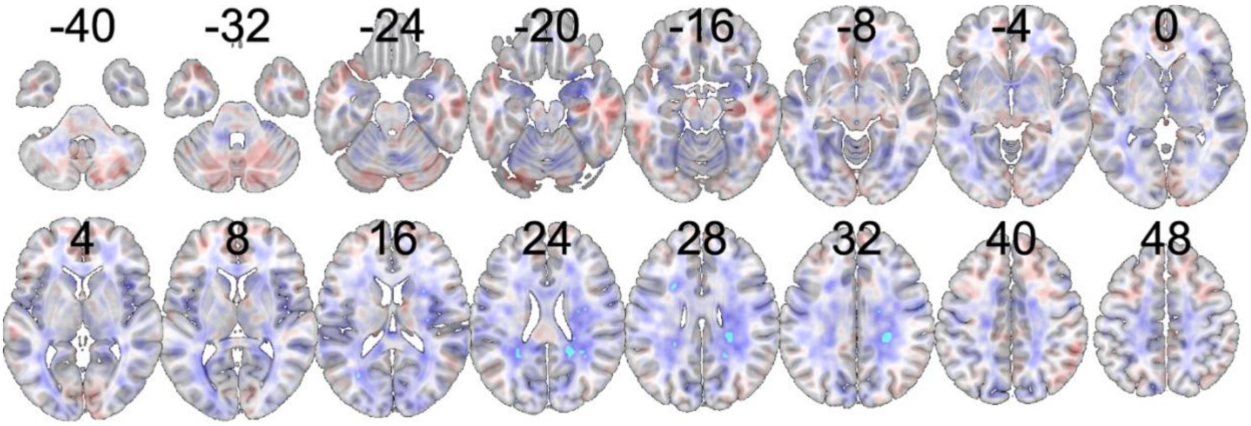

Red denotes regions where placebo-related increases in pain-related processing (in Hedge's *g*) were larger in the "Conditioning + Suggestions" studies (14 independent studies), or equivalently regions where placebo-related decreases in pain related processing were smaller, than in the "Suggestion Only" studies (6 independent studies). Voxels surpassing the statistical threshold of $p < .001$ in two-sided z-tests, uncorrected for multiple comparisons, are highlighted in light blue (range *g*: [-0.45; +0.45]. Only voxels in the negative effect range surpassed the threshold. No voxels reached statistical significance when correcting for FWER.

Please note that the present study was not intended, nor powered for between-study comparisons as the one shown above. Moreover, the 20 studies involved in this analysis cluster in terms of placebo-, pain- and imaging- related features, e.g. the type of placebo induction is not balanced with respect to the stimulus modality (heat, mechanical, visceral, laser, electrical). A simple between-study group comparison as this one may thus be confounded by correlated properties across studies. The question of study-level moderators may better be addressed by a hierarchical meta-regression in subsequent studies.

Supplementary Figure 11: cerebral activity correlating with behavioral placebo analgesia at peak voxels

A right thalamus (R) (prefrontal /premotor subportion)

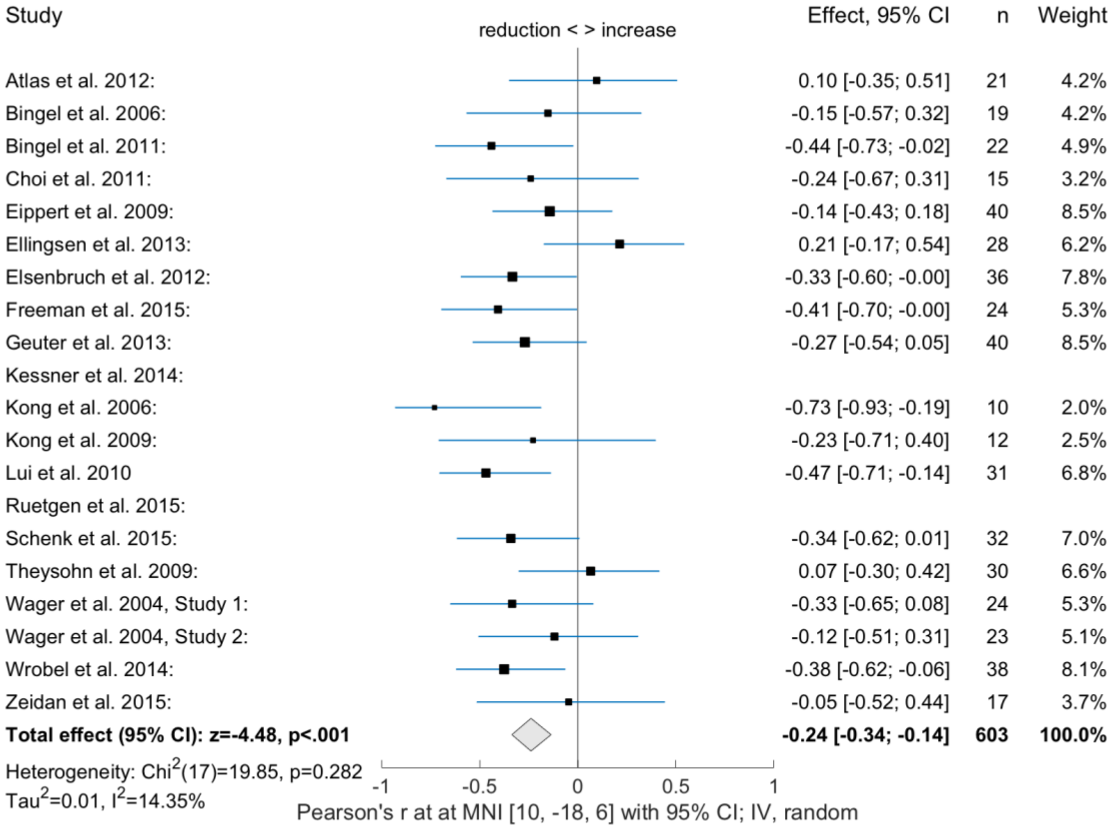

Source data (results as 3d-volumes) are provided at https://osf.io/n9mb3/.

B left thalamus (prefrontal / temporal sub-portion)

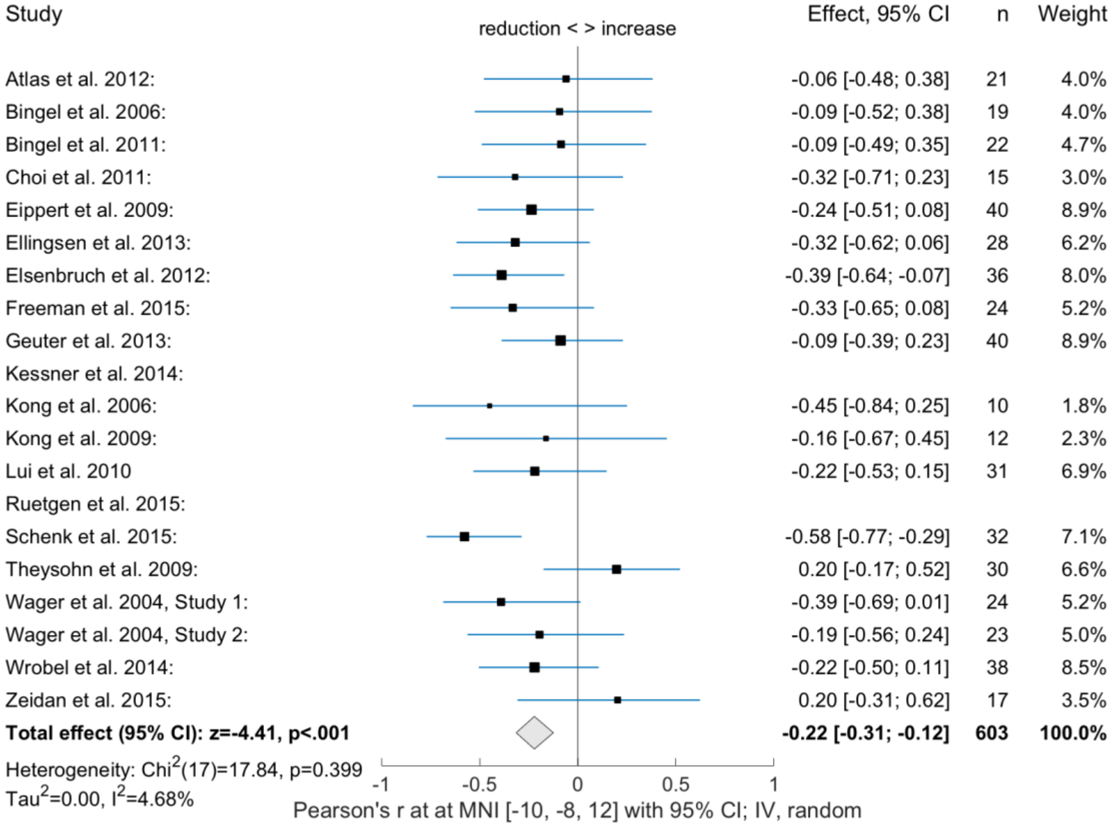

Source data (results as 3d-volumes) are provided at https://osf.io/n9mb3/.

C anterior cingulate gyrus / paracingulate gyrus

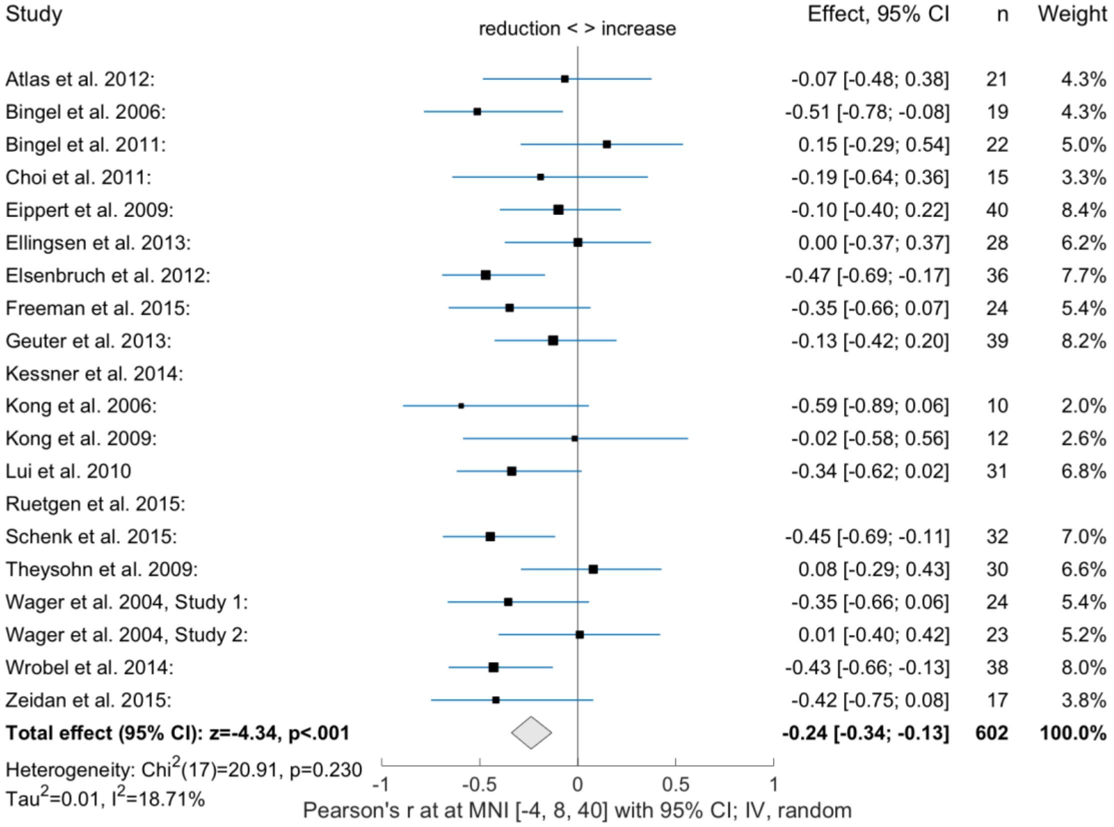

Source data (results as 3d-volumes) are provided at https://osf.io/n9mb3/.

D inferior frontal gyrus, pars triangularis

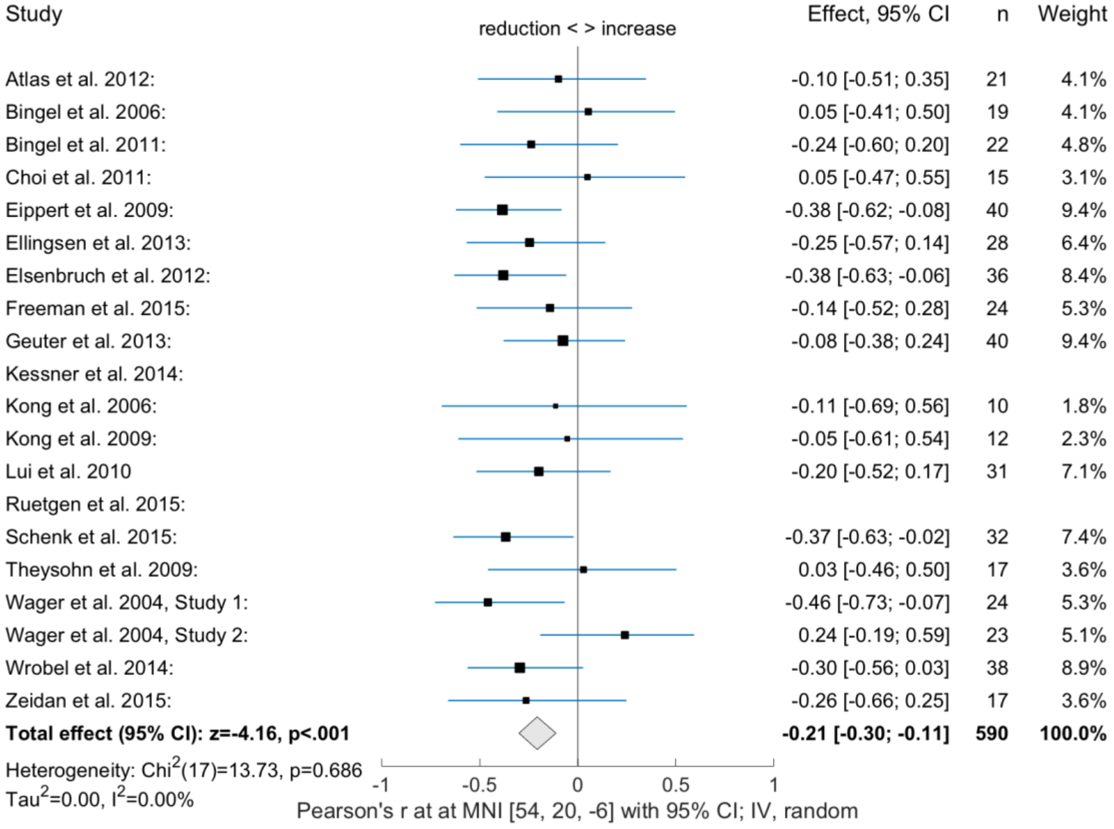

Source data (results as 3d-volumes) are provided at https://osf.io/n9mb3/.

E precentral gyrus / posterior cingulate gyrus

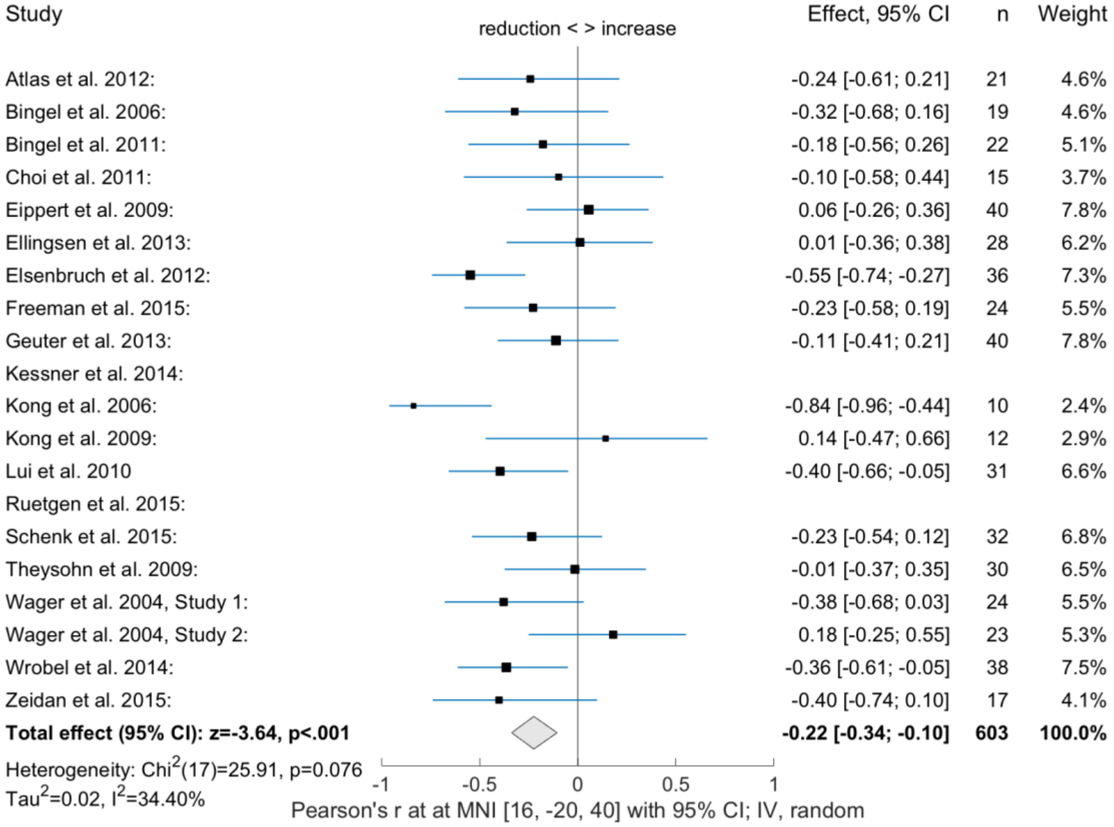

Source data (results as 3d-volumes) are provided at https://osf.io/n9mb3/.

F supplementary motor area / superior frontal gyrus

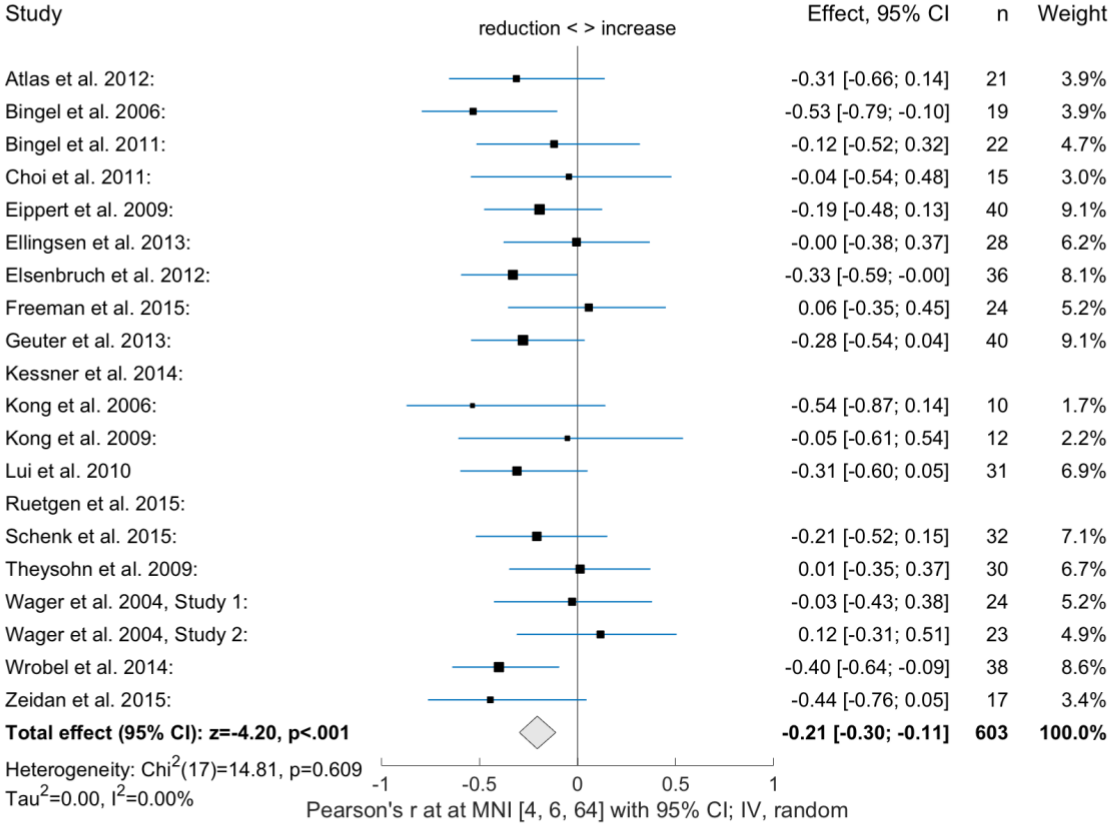

Note that *r*-values were transformed to and from Fisher's *Z* for analysis, resulting in asymmetric confidence intervals. Source data (results as 3d-volumes) are provided at https://osf.io/n9mb3/.

Supplementary Figure 12: between-study heterogeneity versus correlation of cerebral and behavioral placebo effects

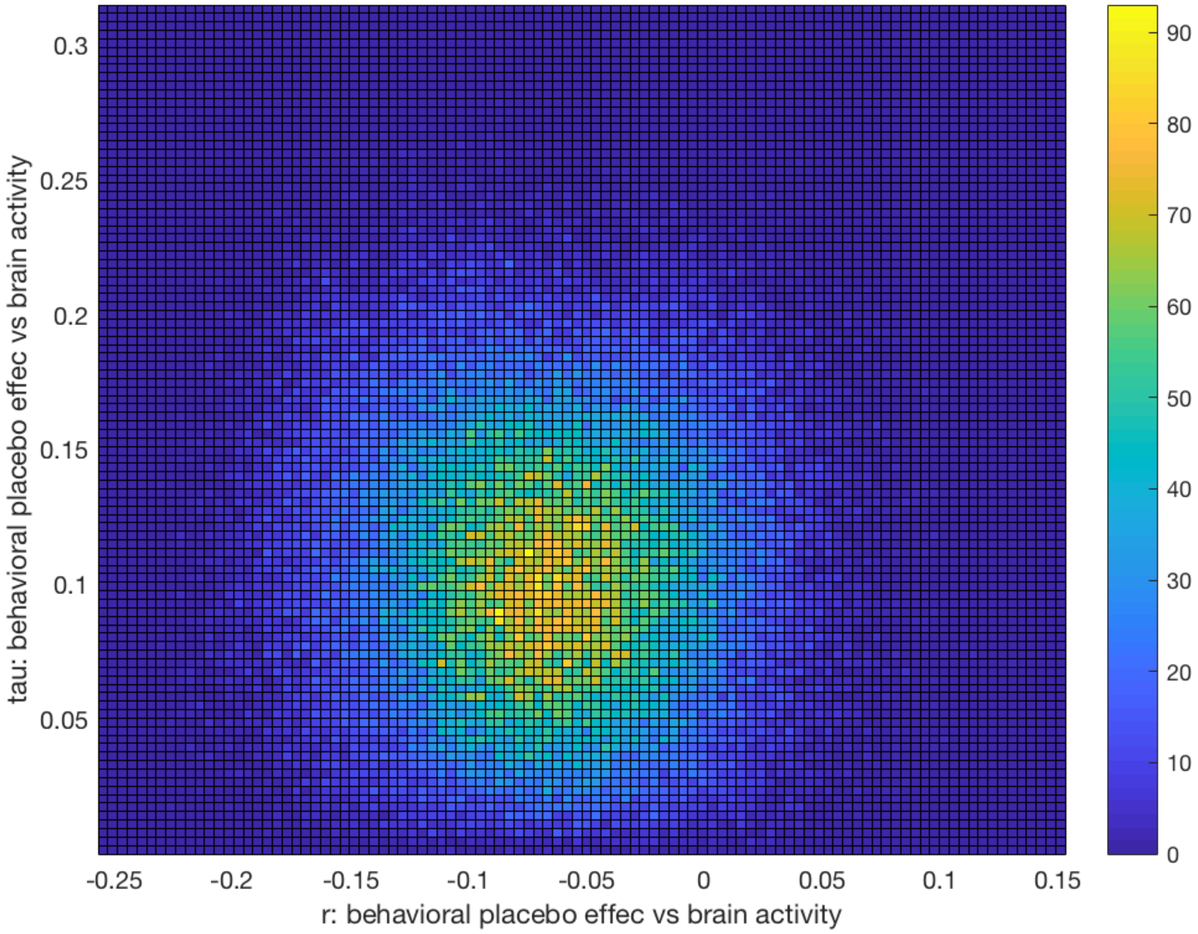

Across all brain-voxels (*n* = 191119 voxels, full sample sans between-group studies), there was a negligible, negative, statistically significant correlation (*r* = -.057, 95% CI [-.061, -.053], *p* < .001) between effects of placebo treatment and between-study heterogeneity estimate τ. Voxels where $\tau = 0$ (49% of voxels) were excluded from the plot, but not the correlation analysis, for illustration purposes. Source data (results as 3d-volumes) are provided at https://osf.io/n9mb3/.

Supplementary Figure 13: a comparison of placebo-related brain activation changes with regions contributing to the NPS.

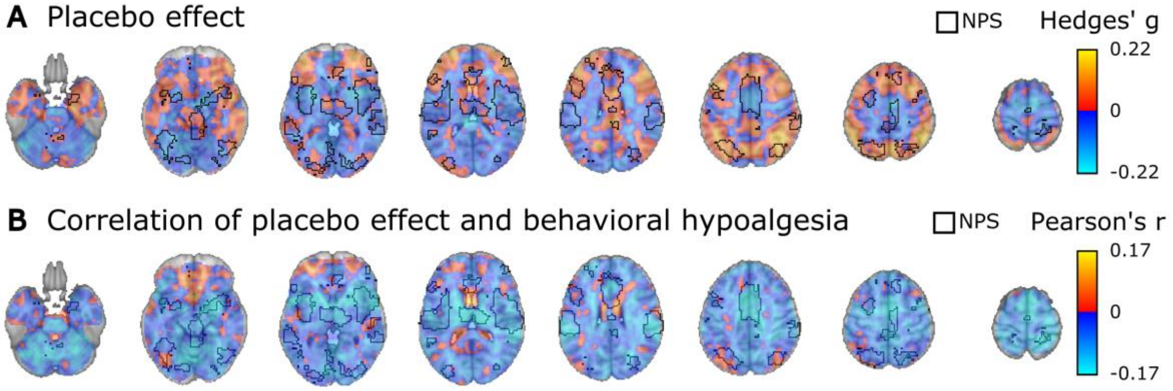

The outline of areas comprising the NPS versus (A) Placebo induced changes in pain-related activity (n = 543 to 603 individuals from 17 to 20 independent studies per voxel) and (B) correlations of behavioral placebo analgesia and changes in pain-related brain activity (n = 384 to 460 individuals from 15 to 18 independent studies per voxel). Note that the outlines above do not differentiate between NPS regions with a positive (more activity indicates more pain) and a negative (more activity indicates less pain) weighting.

Three dimensional coronal slices are equidistantly distributed from *y* = 60 to -68 mm. Axial slices range equidistantly from *z* = -22 to 42 mm. Custom coordinates for sagittal slices is displayed in mm and were chosen to highlight important areas of activation. Source data (results as 3d-volumes) are provided at https://osf.io/n9mb3/.

Supplementary Figure 14: atlases used for similarity-based analysis of brain activity

A whole-brain cortical networks of functional connectivity (Yeo et al. 2011)

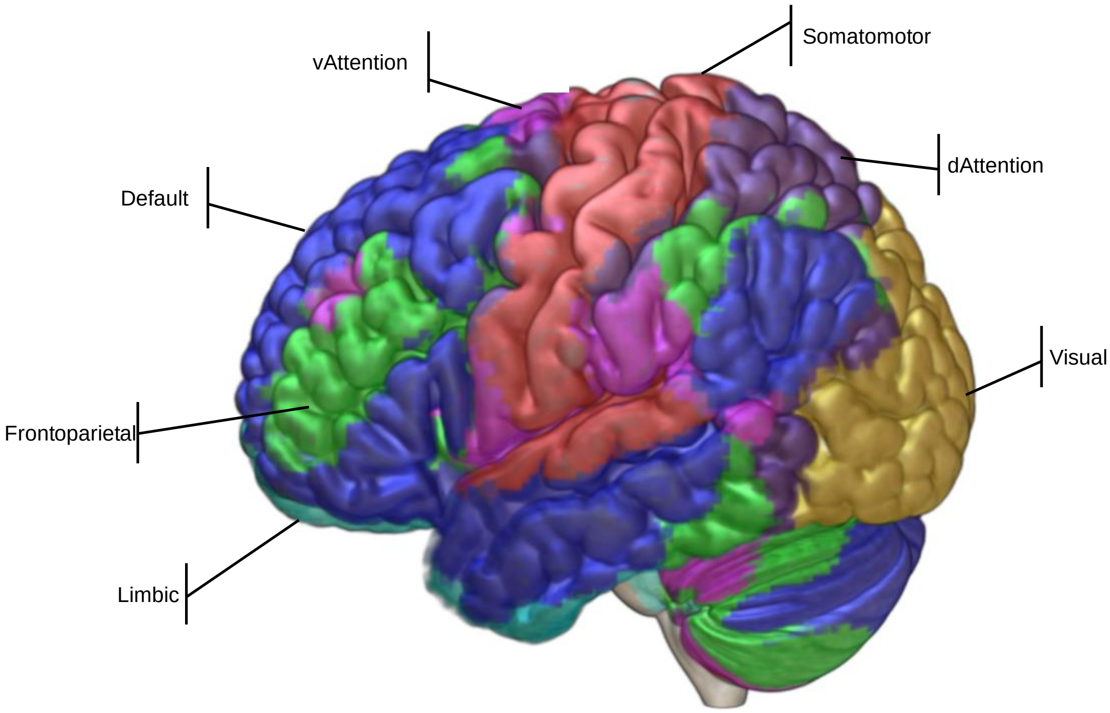

B insular sub-regions (Faillenot et al. 2017)

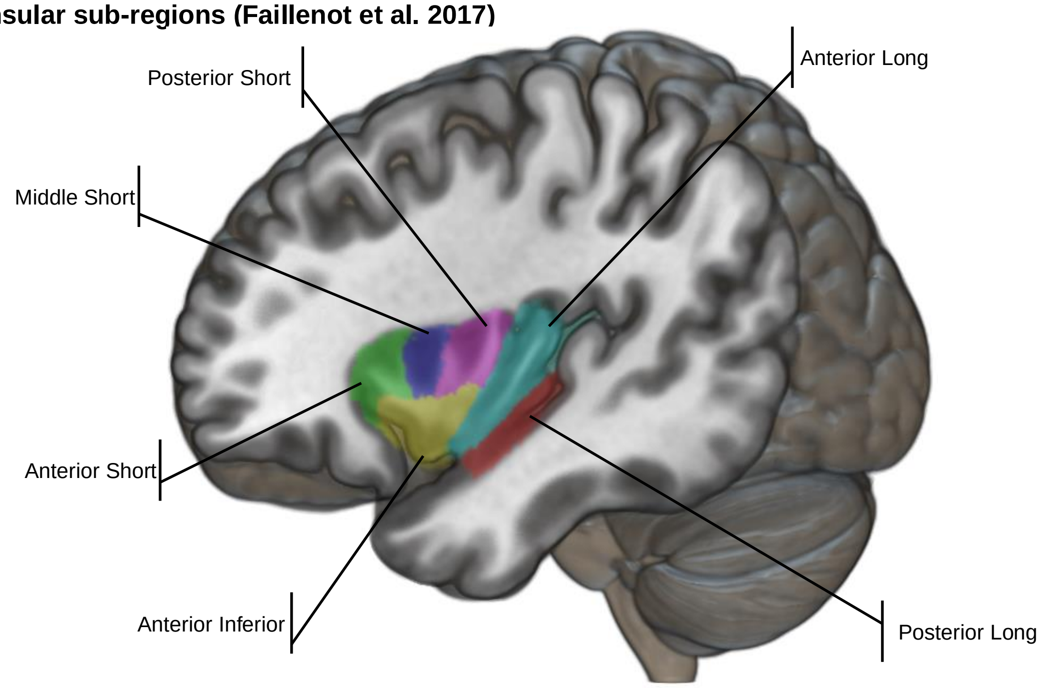

C thalamic nuclei (Krauth et al. 2010)

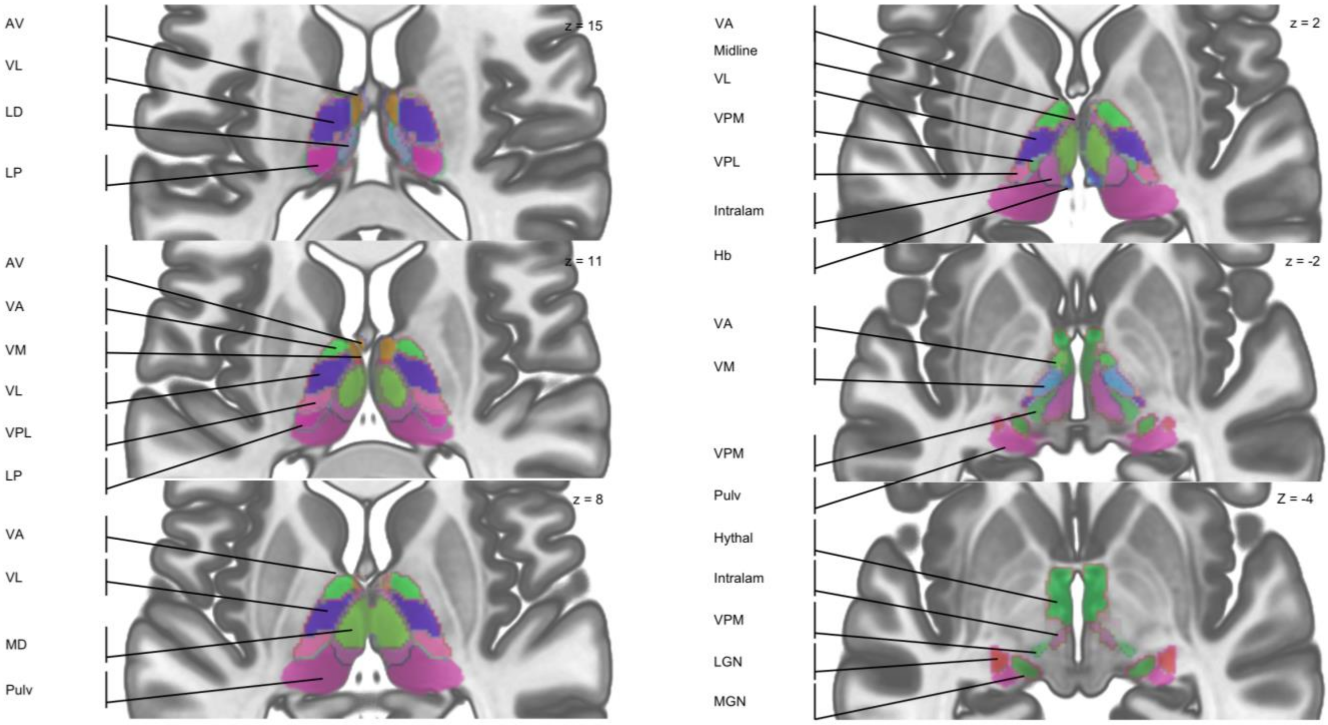

Abbreviations: Hythal: Hypothalamus, Hb: Habenular, AV anterior ventral:, AM anterior medial:, MD: mediodorsal, VM: ventral medial, VA ventral anterior:, LP: lateral posterior, VL: ventral lateral, LD: lateral dorsal, Intralam: intralaminary, VPM: ventral posterior medial, VPL: ventral posterior lateral, MGN: Medial Geniculate Nucleus, LGN: Lateral Geniculate Nucleus, Pulv: Pulvinar.

Supplementary Tables

Supplementary Table 1: study screening, eligibility checking, and retrieval

#	First author	Year	PMID	Source	Comment	*n*
**Eligible, included**						
1	Atlas	2012	22674280	MA	Included	21
2	Bingel	2006	16364549	MS	Included	19
3	Bingel	2011	21325618	MA	Included	22
4	Choi	2011	21546858	MS	Included	15
5	Eippert	2009	19709634	MS	Included	40
6	Ellingsen	2013	24127578	MS	Included	28
7	Elsenbruch	2012	22136749	MS	Included	36
8	Freeman	2015	25776211	Rec	Included	24
9	Geuter	2013	23201367	MS	Included	48
10	Lui	2010	20943318	MS	Included	33
11	Kessner	2014	25275613	MS	Included	39
12	Kong	2006	16407533	MS	Included	16
13	Kong	2009	19159691	MS	Included	12
14	Rütgen	2015	26417092	Rec	Included	102
15	Schenk	2014	24076046	Rec	Included	32
16	Theysohn	2014	25346054	MS	Included	33
17	Wager (Study I)	2004	14976306	MS	Included	25
18	Wager (Study II)	2004	14976306	MS	Included	24
19	Wrobel	2014	24796219	MS	Included	44
20	Zeidan	2015	26586819	Rec	Included	20
**Eligible, not available**						
21	Craggs	2014	24412799	MS	Responded, data unavailable	15
22	Lee	2012	22541443	MS	No response	34
23	Lu	2010	19962240	MS	No response	14
24	Nemoto	2007	17287994	MA	No response	10
25	Petrovic	2002	11834781	MA	Responded, data unavailable	9
26	Price	2007	16963184	MS	Responded, data unavailable	9
27	Sevel	2015	25659463	MS	Responded, data unavailable	24
28	Watson	2009	19523766	MS	Responded, data unavailable	11
**Eligible, published after study-search**						
29	Fehse	2015	25933389	PS	Not sought	30
30	Schenk	2017	28883019	PS	Not sought	48
31	van der Meulen	2017	28338955	PS	Not sought	30
32	Gollub	2018	29325883	PS	Not sought	45
33	Linnman	2018	29255671	PS	Not sought	18
34	Yue	2018	29025005	PS	Not sought	25
**Assessed for eligibility, not eligible**						
35	Chae	2009	19533753	MS	Placebo & pain conditions not separable	na
36	Craggs	2007	17904390	MS	Re-analysis of 16963184	na
37	Craggs	2008	18804916	MS	Re-analysis of 16963184	na
38	Eippert	2009	19833962	MS	Spinal	na
39	Jensen	2014	25452576	MS	No treatment context (cued expectancy)	na
40	Kotsis	2012	22747652	MS	Re-analysis of 22136749	na
4	Leech	2013	24093551	MS	No experimental pain (cough)	na
42	Huber	2013	23664683	MS	Re-analysis of 20943318	na
43	Petrovic	2010	20399560	MS	Re-analysis of 11834781	na
44	Schmid	2015	24833636	MS	Re-analysis of 25346054	na
45	Wager	2011	21228154	MS	Re-analysis of 14976306	na
46	Zhang	2013	23123362	MS	No experimental pain in fMRI	na
**Screened, not eligible**						
47	Amanzio	2013	22125184	MS	Review/comment	na

48	Beauregard	2009	19023697	MS	Review/comment	na
49	Benedetti	2007	17379417	MS	Review/comment	na
50	Berna	2011	21815494	MS	Review/comment	na
51	Bingel	2010	20376600	MS	Review/comment	na
52	Blom	2011	21734437	MS	No experimental placebo, no fMRI	na
53	Büchel	2014	24656247	MS	Review/comment	na
54	Colloca	2008	17960416	MS	Review/comment	na
55	Columbo	2015	25758451	MS	No experimental placebo, no fMRI	na
56	Dalakas	1995	7611640	MS	No experimental placebo, no fMRI	na
57	Dobrila-Dintinjana	2011	22220463	MS	Review/comment	na
58	Dukart	2014	24379394	MS	No experimental placebo	na
59	Gamus	2015	25796668	MS	Review/comment	na
60	Ghahreman	2011	21539702	MS	No experimental placebo, no fMRI	na
61	Grabowski	2010	20677441	MS	Review/comment	na
62	Gupta	2011	21250799	MS	No experimental placebo, no fMRI	na
63	Hashmi	2012	22531485	MS	No experimental pain	na
64	Hashmi	2012	22985900	MS	No experimental pain	na
65	Höller	2009	19573501	MS	Review/comment	na
66	Howell	2010	20839687	MS	No experimental placebo, no fMRI	na
67	Hróbjartsson	2011	21524568	MS	Review/comment	na
68	Johnson	2004	15134003	MS	Review/comment	na
69	Khalili-Mahani	2015	25554429	MS	No experimental pain in fMRI	na
70	Kong	2007	18019605	MS	Review/comment	na
71	Li	2010	21280461	MS	Review/comment	na
72	Li	2014	24817188	MS	No experimental placebo	na
73	Lidstone	2007	17334853	MS	Review/comment	na
74	Lu	2011	21751434	MS	Review/comment	na
75	Martini	2015	25523008	MS	No fMRI	na
76	Miura	2013	23711332	MS	No experimental pain	na
77	Murray	2013	23880289	MS	Review/comment	na
78	Nandhagopal	2008	18413571	MS	Review/comment	na
79	Petersen	2014	25281929	MS	No fMRI	na
80	Petrovic	2005	15953423	MS	No experimental pain	na
81	Qiu	2009	19784082	MS	Review/comment	na
82	Rainville	2006	16513275	MS	Review/comment	na
83	Rigatelli	2008	18759545	MS	Review/comment	na
84	Ritter	2014	24672009	MS	No experimental placebo	na
85	Sant'Anna	2014	25372920	MS	No experimental placebo	na
86	Sarinopoulos	2006	16472720	MS	No experimental pain	na
87	Scott	2007	17640532	MS	No experimental pain in fMRI	na
88	Scott	2008	18250260	MA	Pharmacological PET	na
89	Stein	2012	22959599	MS	No experimental placebo	na
90	Su	2010	21290837	MS	Review/comment	na
91	Theis	2004	15354245	MS	Review/comment	na
92	Wager	2007	17578917	MA	Pharmacological PET	na
93	Wager	2013	24761154	MS	Review/comment	na
94	Werndle	2015	24819624	MS	No experimental placebo	na
95	Wiech	2014	25093555	MS	No fMRI	na
96	Wu	2014	24268723	MS	No experimental placebo	na
97	Xu	2014	25069206	MS	Review/comment	na
98	Yelle	2009	19692600	MS	No experimental placebo	na
99	Yilmaz	2010	20817354	MS	No experimental placebo	na
100	Yu	2014	24578196	MS	No experimental pain	na
101	Zhang	2011	21332487	MS	No experimental pain	na
102	Zubieta	2009	19338509	MS	Review/comment	na

The *n* shown for eligible studies refer to participants that completed testing according to the original manuscripts. Abbreviations: fMRI, functional Magnetic Resonance Imaging; MS, study identified in an initial medline search; na, not assessed; MA, study identified in previous meta-analyses; PS, study identified i post-hoc search; Rec, study added late after recommendaton by collaborators during data acquisition, Sample identical with [1].

Supplementary Table 2: included studies: design, demographics, & heat stimulation

#	First Author	year	*n*	Pain type	Pain location	Stimulus duration (s)	Stimulus intensity	Pain rating
1	Atlas	2012	21	heat	L forearm (v)	10	"[…]applied temperatures were calibrated to elicit levels of low pain (VAS rating = 2;M = 41.16°C, SD = 2.64) and high pain(VASrating = 8; M = 47.05°C, SD = 1.69)[…]"	"[...] continuous, numerically anchored visual analog scale (VAS) from 0 to 8 (0, no sensation; 1, nonpainful warmth; 2, low pain; 5, moderate pain; 8, maximum tolerable pain)."
2	Bingel	2006	19	laser	L & R hand (d)	0.001	"[…] laser pain stimuli of 600 mJ each were applied to the respective hand every 6–8 s"	"[...] another vocal command ('rating') prompted the subject to rate the average sensation for the last four painful stimuli with hand signs on the numerical rank scale (NRS) ranging from 0 (no sensation) to 4 (maximum pain used in the experiment)."
3	Bingel	2011	22	heat	R calf (d)	6	"For each participant, the temperature of the thermode was adjusted to produce a pain intensity rating of 70 on a VAS, where 0 corresponds to "no pain" and 100 to "unbearable pain." This temperature was delivered during all runs."	"[…] pain intensity rating performed on a VAS (100 parts; endpoints labeled with no pain and unbearable pain)."
4	Choi	2011	15	electri-cal	L hand (d)	15	"Each participant received the same level of electrical stimulation (2 Hz, 20 mA, duration: 15 s) during fMRI scanning […]"	"Ratings were assessed using a Numerical Rating Scale ranging from 0 to 100 (0 = no pain or anxiety; 100 = maximum imaginable pain or anxiety)."
5	Eippert	2009	40	heat	L forearm (v)	17	"Importantly, in both sessions subjects were stimulated with the same temperature (equivalent to 60 on the VAS)."	"[…](VAS; 100 parts; endpoints labeled with "no pain" and "unbearable pain")[…]"
6	Ellingsen	2013	28	heat	L forearm (d)	10	"A moderately painful temperature, which was selected for each participant before the first fMRI session (5 on a numeric rating scale, NRS, with anchors 0 = no pain; 1 = pain threshold; 10 = intense pain), was used in both fMRI sessions (mean temperature = 47.1 ± 0.73 °C)."	"Hedonic Ratings. A VAS (−5to +5) with anchors "unpleasant" and "pleasant" […]"

7	Elsenbruch	2012	36	disten-sion	C rectal	31	“Subjects were prompted to rate the sensation as follows: 1 = no perception; 2 = doubtful perception; 3 = sure perception; 4 = little discomfort; 5 = severe discomfort, still tolerable; 6 = pain, not tolerable. For repeated distensions in the scanner, the pressure corresponding to a rating of 5 was chosen.”	"Visual analogue scales (VAS) (0 to 100 mm; ends defined as 0: none to 100: very much) were completed after each session to quantify subjective pain[...]"
8	Freeman	2015	24	heat	R forearm (v)	7	“[…] temperature was moderate [10–11 out of 20 rating] on the final 6 regions demarcated on the volar forearm.”	"[...]Gracely Scales (0–20) (Gracely et al., 1978a, 1978b) that they [the participants] would use to rate their pain[...]"
9	Geuter	2013	40	heat	L forearm (v)	16	“[…]identical stimuli (VAS 60) were applied on placebo and control patches, respectively (15 on each patch)[..]”	"[...] subjects rated their pain intensity on a computerized visual analogue scale (VAS) ranging from 0 to 100. The scale was anchored with “no pain” and “unbearable pain”. Subjects were instructed to rate “unbearable pain” only in case they had to lift the thermode because of too intense pain."
10	Kessner	2014	39	heat	L forearm (v)	16	“In all participants, a stimulus intensity of VAS 50 was applied at the ointment treatment site and of VAS 80 at the untreated site (15 stimuli each).”	“"The participants were asked to rate each pain stimulus on a Visual Analog Scale (VAS, [100 parts; endpoints labeled as “no pain” and “unbearable pain”])."”
11	Kong	2006	10	heat	R forearm (v)	5	“Temperatures that elicited subjective intensity ratings in the low pain range (8–11; the mild to moderate range on the 0–20 Sensory Box scale) and high pain range (14–17; the strong to intense range on the 0–20 Sensory Box scale) were selected for each subject."	“[...] teach the subjects to rate the stimuli using the Sensory Box and Affective Box 0–20 scales (Gracely et al., 1978a,b, 1979).”
12	Kong	2009	12	heat	R forearm (v)	12	“[…] temperatures eliciting subjective intensity ratings in the LOW pain range (~5; which indicates weak on the 0–20 Sensory Scale) and HIGH pain range (~15; strong) were selected for each individual[…]” “stimulus temperatures and the corresponding subjective sensory ratings (mean ± SD) were 48.1 ± 1.1 °C and 14.5 ± 1.6 for HIGH pain; 45.1 ± 1.6 °C and 5.0 ± 2.7 for LOW pain”	“Gracely Sensory and Affective scales (Gracely et al., 1978a,b) were used to measure subjective pain ratings.”

13	Lui	2010	31	laser	L or R foot (v)	~0.05	“An ascending series of stimuli were delivered in steps of 0.5 J, starting from very low intensities (0.5 J, below warmth threshold) until a mild-to-moderate pain intensity was achieved for each subject.”	“[…]volunteers had to rate the perceivedpain intensity,byrotatingaknobwhichmovedacursor on a computerized visual analogue scale (VAS), anchored at 0 = no pain, and 100 = worst imaginable pain.”
14	Rütgen	2015	102	electri-cal	L hand (d)	0.5	“In the fMRI experiment, average stimulation intensity was 0.16 mA (SD 0.15) for nonpainful sensations and 0.74 mA (SD 0.59) for painful sensations.”	“After stimulation of themselves, participants rated their own pain (self-directed pain ratings), using the question “How painful was this stimulus for you?” on a seven-point rating scale ranging from “not at all” to “extremely painful.””
15	Schenk	2014	32	caps + heat	L & R forearm (v)	20	“Temperature calibration was per- formed to elicit a pain level of approximately 6 on a VAS (0–10)”, “The average temperature corresponding to a VAS rating of 6 was 39.8 ± 2.9°C on capsaicin-pretreated skin.”	“[…] subjects rated their perceived pain intensity on a VAS scale (0–10, end points labeled with “no pain at all” and “unbearable pain”, 10 seconds).”
16	Theysohn	2014	30	disten-sion	C rectal	16.8	“In all three sessions, subjects received rectal distensions at a pressure just below the individual pain threshold[…]”	“[…] distension-induced pain (after each distension) VAS scales, with ends defined as ‘no pain/tension’ and ‘maximal pain/tension’. For analyses, all responses were quantified in mm from ‘0’ to ‘100’.”
17	Wager[A]	2004	24	electri-cal	R forearm (v)	6	Mild shock intensity was defined as the level of the shock just prior to the point at which participants acknowledged pain (mean = 1.44mA, sd = 0.85 mA). Intense shocks were set at the maximum level participants could tolerate (mean = 3.75 mA, sd = 2.34 mA).	“participants rated the intensity of the shock on a 10-point scale” — original pain ratings not available, only placebo-control contrast of ratings
18	Wager[B]	2004	23	heat	L forearm (v)	17	“Two repetitions of 3 temperatures(starting at 45, 47, and 49 degrees Celsius) were administered, and temperatures were adjusted and the test repeated as necessary to find pain levels 2, 5, and 8 for each participant on a 10-point scale (1 was “just painful”, 10 was “unbearable pain”). On all trials, a 20-s thermal stimulation (17 s plateau, 1.5 s ramp up / ramp down to baseline)was followed by a 40-s rest period. Temperatures were 45.4 degrees centigrade	“[…] reported pain levels […] on a 10-point scale (1 = just painful; 10 = unbearable pain)” — original pain ratings not available, only placebo-control contrast of ratings

							on average (sd = 1.1) for Level 2, 47.0 (sd = 0.9) for Level 5, and 48.1 (sd = 1.0) for Level 8."	
19	Wrobel	2014	38	heat	L forearm (v)	17	"[…]placebo and control sites were stimu-lated with the same individually calibrated temperature cor- responding to VAS 60."	"VAS (100 parts; endpoints labeled with 'no pain' and 'unbearable pain')"
20	Zeidan	2015	19	heat	R leg (d)	12	"heat; 49°C + neutral; 35°C" (Figure 1)	"pain intensity and unpleasantness ratings were as- sessed with a 15 cmplastic sliding visual analog scale (VAS) (Price et al., 1994) […]The minimum rating ("0") was designated as "no pain sensation" and "not at all unpleasant," whereas the maximum ("10") was labeled as "most intense pain sensation imag- inable" or "most unpleasant sensation imaginable," respectively."

Abbreviations: [A] Sub-study 1; [B] Sub-study 2; Caps, capsaicin; (d), dorsal; na, not available; (v), ventral.

Supplementary Table 3: included studies: placebo conditions

#	First author	year	Placebo induction	Placebo type	Placebo treatment conditions
1	Atlas	2012	Suggestions	IV-infusion	Within(hidden vs open remifentanil)*
2	Bingel	2006	Suggestions + conditioning	Topical cream	Within(placebo vs control)
3	Bingel	2011	Suggestions + conditioning	IV-infusion	Within(hidden vs open remifentanil)*
4	Choi	2011	Suggestions + conditioning	IV-infusion	Within(high vs low vs no efficacy)
5	Eippert	2009	Suggestions + conditioning	Topical cream	Within(placebo vs control) x Between(naloxone vs saline)
6	Ellingsen	2013	Suggestions	Nasal spray	Within(placebo vs no treatment)
7	Elsenbruch	2012	Suggestions	IV-infusion	Within(high vs low vs no chance of efficacy)
8	Freeman	2015	Suggestions + conditioning	Topical cream	Within(placebo vs nocebo vs control)
9	Geuter	2013	Suggestions + conditioning	Topical cream	Within(expensive high vs cheap low vs no efficacy)
10	Kessner	2014	Conditioning	Topical cream	Between(effective vs ineffective placebo conditioning)
11	Kong	2006	Suggestions + conditioning	Sham acupuncture	Within(placebo vs control)
12	Kong	2009	Suggestions + conditioning	Sham acupuncture	Within(placebo vs control)
13	Lui	2010	Suggestions + conditioning	Placebo TENS	Within(placebo vs control)
14	Rütgen	2015	Suggestions + conditioning	Pill	Between(placebo vs no treatment)
15	Schenk	2014	Suggestions	Topical cream	Within(hidden vs open*) x Within(placebo vs control)
16	Theysohn	2014	Suggestions	IV-infusion	Within(placebo vs control)
17	Wager[A]	2004	Suggestions	Topical cream	Within(placebo vs control)
18	Wager[B]	2004	Suggestions + conditioning	Topical cream	Within(placebo vs control)
19	Wrobel	2014	Suggestions + conditioning	Topical cream	Within(placebo vs control) x Between(haloperidol vs saline)
20	Zeidan	2015	Suggestions + conditioning	Topical cream	Within(placebo vs no treatment)

* In analogy to the other studies open treatment were treated as “placebo conditions” and hidden treatment conditions as “control condition”.
Abbreviations: between, between-group factor; [A] Sub-study 1; [B] Sub-study 2; IV, intravenous; within, within subject factor.

Supplementary Table 4: included studies: functional neuro imaging acquisition characteristics

#	First author	year	Field Strength (Tesla)	TR (ms)	TE (ms)	Resolution (mm)	Images/participant
1	Atlas	2012	1.5	2000	34	3.5*3.5*4.0	1980
2	Bingel	2006	1.5	2600	40	3.3*3.3*4.0	976
3	Bingel	2011	3	3000	30	3.5*3.5*3.0	ø 734
4	Choi	2011	3	3000	30	3.8*3.8*4.0	300
5	Eippert	2009	3	2620	26	2.0*2.0*3.0	ø 658
6	Ellingsen	2013	3	2000	30	3.0*3.0*3.3	510
7	Elsenbruch	2012	1.5	3100	50	3.8*3.8*3.3	591
8	Freeman	2015	3	2000	40	3.1*3.1*5.0	unknown
9	Geuter	2013	3	2580	26	2.0*2.0*3.0	ø 1137
10	Kessner	2014	3	2580	26	2.0*2.0*3.0	662
11	Kong	2006	3	2000	40	3.1*3.1*5.0	unknown
12	Kong	2009	3	2000	40	3.1*3.1*5.0	unknown
13	Lui	2010	3	3014	35	1.9*1.9*3.5	648
14	Rütgen	2015	3	1800	33	1.5*1.5*2.0	ø 507
15	Schenk	2014	3	2580	26	2.0*2.0*2.0	1260
16	Theysohn	2014	1.5	2400	26	2.6*2.6*3.0	617
17	Wager[A]	2004	3	1800	22	3.8*3.8*5.0	600
18	Wager[B]	2004	3	1500	20	3.0*3.0*4.0	640
19	Wrobel	2014	3	2580	25	2.0*2.0*3.0	ø 655
20	Zeidan*	2015	3	NA	NA	3.4*3.4*6.0	8

All studies obtained blood-oxygenation-dependent (BOLD) signal using echo-planar imaging (EPI) variants. except for one study (*) using arterial spin labeling (ASL). Image number represents the number of volumes per participant used in the original analysis; for studies with varying imaging duration average (ø) images per participant are reported. All information was obtained from the original publications and (where available) from analysis files (e.g. SPM.mat or design.mat).
Abbreviations: [A] Sub-study 1; [B] Sub-study 2; NA, not applicable; TR repetition time; TE echo time.

Supplementary Table 5: included studies: pre-processing and first-level analysis of neuroimages

#	First author	Year	Software	Slice timing	Spatial smoothing (mm)	Temporal high-pass filter (s)	Other filters	Image type	Modeled pain duration (s)	HRF	Nuisance regressors	Parametric modulators
1	Atlas	2012	SPM5	Yes	8*8*8	180	no	beta	14.2	custom	motion + outliers	expectation + remifentanil
2	Bingel	2006	SPM2	no	8*8*8	128	no	con	Event	canonical	no	temp derivative
3	Bingel	2011	SPM5	yes	8*8*8	128	no	beta	6.0	canonical	no	no
4	Choi	2011	SPM8	no	5*5*5	50	no	beta	15.0	canonical	no	TD
5	Eippert	2009	SPM5	yes	8*8*8	128	no	con	10.0 early, 10.0 late	canonical	no	no
6	Ellingsen	2013	FSL	no	5*5*5	120	ICA	con	10.0	gamma	no	no
7	Elsenbruch	2012	SPM5	no	9*9*9	140	LP	beta	31.0	canonical	no	no
8	Freeman	2015	SPM8	no	8*8*8	128	no	con	7.0	canonical	no	no
9	Geuter	2013	SPM8	no	6*6*6	128	no	con	10.0 early, 10.0 late	canonical	motion + CRF & WM signal	no
10	Kessner	2014	SPM8	yes	8*8*8	128	no	beta	10.0 early, 10.0 late	canonical	no	no
11	Kong	2006	SPM2	no	8*8*8	128	no	con	5.0	canonical	no	no
12	Kong	2009	SPM2	no	8*8*8	128	no	con	7.0	canonical	no	no
13	Lui	2010	SPM5	yes	4*4*8	128	no	con	Event	canonical	no	TD + ratings
14	Rütgen	2015	SPM12	yes	6*6*6	128	no	con	4.4	canonical	motion	no
15	Schenk	2014	SPM8	no	6*6*6	128	no	beta	20.0	canonical	no	no
16	Theysohn	2014	SPM8	no	8*8*8	120	LP	beta	16.8	canonical	no	no
17	Wager[A]	2004	SPM99	yes	6*6*6	128	LP	con	20.0	canonical	no	no
18	Wager[B]	2004	SPM99	yes	9*9*9	100	WM mask	beta	6.0	none	movement	no
19	Wrobel	2014	SPM8	yes	8*8*8	128	no	beta	10.0 early, 10.0 late	canonical	no	no
20	Zeidan*	2015	FSL	NA	9*9*9	NA	NA	con	12.0	NA	movement + WM signal	no

All studies obtained blood-oxygenation-dependent (BOLD) signal using echo-planar imaging (EPI) variants, except for * who obtained arterial spin labeling (ASL). For spatial smoothing a gaussian kernel filter was used in all studies, full-width-half-maximum kernel is provided in mm. All information was obtained from the original publications and (where available) from analysis files (e.g. SPM.mat or design.mat). Source data (results as 3d-volumes) are provided at https://osf.io/n9mb3/.

Abbreviations: [A] Sub-study 1; [B] Sub-study 2; CRF, cerebrospinal fluid; hrf, hemodynamic response function; ICA, independent-component analysis used for temporal noise filtering; LP, temporal low pass filter; NA, not applicable; TD, temporal derivative; TE, echo time; temo, temporal; TR, repetition time; WM, white matter.

Supplementary Table 6: experimental conditions selected for full and conservative analysis

#	First Author	year	Full sample	Conservative sample
1	Atlas	2012	Hidden vs open remifentanil; sum(pain stimulation, remifentanil effect, expectation period)	Hidden vs open remifentanil; sum(pain stimulation, remifentanil effect, expectation period)
2	Bingel	2006	Control vs placebo; mean(left & right side)	Control vs placebo; mean(left & right side)
3	Bingel	2011	Remifentanil hidden vs open	**Excluded due to fixed testing sequence**
4	Choi	2011	No treatment vs mean(low, high efficacy placebo)	No treatment vs mean(low, high efficacy placebo)
5	Eippert	2009	Control vs placebo; mean(early, late pain), saline and naloxone group	Control vs placebo; mean(early, late pain), saline and naloxone group
6	Ellingsen	2013	Placebo vs no treatment; painful heat	Placebo vs no treatment; painful heat
7	Elsenbruch	2012	No (0%) vs certain (100%) placebo	No (0%) vs certain (100%) placebo
8	Freeman	2015	Control vs placebo	Control vs placebo
9	Geuter	2013	Control vs mean (weak, strong placebo); mean(early, late pain)	Control vs mean (weak, strong placebo); mean(early, late pain)
10	Kessner	2014	Negative vs positive experience group (placebo site)	Negative vs positive experience group (placebo site)
11	Kong	2006	Control vs placebo (high pain)	Control vs placebo (high pain)
12	Kong	2009	Control vs placebo (high pain)	Control vs placebo (high pain)
13	Lui	2010	Red vs green cue signifying sham TENS off/on	Red vs green cue signifying sham TENS off/on
14	Rütgen	2015	No treatment vs placebo group	**Excluded due to responder selection**
15	Schenk	2014	mean(control, hidden lidocaine) vs mean(placebo, open lidocaine)	mean(control, hidden lidocaine) vs mean(placebo, open lidocaine)
16	Theysohn	2014	No (0%) vs certain (100%) placebo	No (0%) vs certain (100%) placebo
17	Wager[A]	2004	Control vs placebo*	Control vs placebo*
18	Wager[B]	2004	Control vs placebo*	**Excluded due to responder selection**
19	Wrobel	2014	Control vs placebo; mean(early pain, late pain), (saline & haloperidol group)	Control vs placebo; mean(early pain, late pain), (saline & haloperidol group)
20	Zeidan	2015	Control vs placebo*; placebo group	**Excluded due to fixed testing sequence and different imaging modality**

For studies marked with an asterisk (*) imaging data were only available as separate contrasts for pain activation and placebo conditions, which could not be re-combined post-hoc. Consequently the within-subject correlations necessary to estimate Hedges' g_{rm} could not be obtained. We therefore imputed the mean correlation observed across all other within-subject studies in these cases.
Abbreviations: [A] Sub-study 1; [B] Sub-study 2.

Supplementary Table 7: risk of bias assessment according to the Cochrane risk-of-bias assessment tool:

Type of bias:			Selection	Performance	Detection	Attrition			Testing Sequence	
#	first author	date	allocation to treatment	blinding of subjects and treatment providers	analyst blinding	subjects available / entered testing (%)	subjects available / completed testing (%)	subjects available / included in original analysis (%)	sequence of placebo sessions per protocol	% of participants where control was tested before placebo
1	Atlas	2012	WI-subject	No blinding of subjects and treatment providers	No blinding of analysts, choice of pre-processing and modelling approach may affect whole-brain summary images	87.5	100.0	100.0	balanced	42.9
2	Bingel	2006	WI-subject			95.0	100.0	100.0	alternating	50.0
3	Bingel	2011	WI-subject			95.7	100.0	100.0	pre-post	100.0
4	Choi	2011	WI-subject			100.0	100.0	100.0	?	?
5	Eippert	2009	WI-subject			83.3	100.0	100.0	balanced	55.0
6	Ellingsen	2013	WI-subject			93.3	100.0	100.0	balanced	53.6
7	Elsenbruch	2012	WI-subject			100.0	100.0	100.0	balanced	55.6
8	Freeman	2015	WI-subject			63.2	100.0	100.0	alternating	50.0
9	Geuter	2013	WI-subject			76.9	83.3	100.0	balanced	46.3
10	Kessner	2014	randomization list			97.5	100.0	100.0	balanced	48.6
11	Kong	2006	WI-subject			41.7	62.5	62.5	alternating	50.0
12	Kong	2009	WI-subject			?	?	100.0	alternating	50.0
13	Lui	2010	WI-subject			86.1	93.9	100.0	alternating	50.0
14	Rütgen	2015	responder selection			85.0	100.0	100.0	between-group	NA
15	Schenk	2014	WI-subject			82.1	100.0	100.0	balanced	53.1
16	Theysohn	2014	WI-subject			83.3	90.9	100.0	balanced	60.0
17	Wager[A]	2004	WI-subject			96.0	96.0	100.0	balanced	?
18	Wager[B]	2004	responder selection			95.8	95.8	100.0	balanced	?
19	Wrobel	2014	WI-subject			76.0	86.4	100.0	balanced	42.1
20	Zeidan	2015	WI-subject			85.0	85.0	89.5	pre-post	100.0
Total:			-	-	-	84.4[1]	95.2[1]	98.7	-	54.1[1]

Red cells denote parameters indicating high risk of bias, yellow cells unknown risk of bias and green cells low risk

Abbreviations: ?, unknown; [A] Sub-study 1; [B] Sub-study 2; [1] excluding studies with unknown values; NA not applicable; NPS, neurologic pain signature; WI-subject, within-subject study design

Supplementary Table 8: clusters showing a significant negative correlation between brain activity and behavioral placebo analgesia — conservative sample (sans between-subject studies, high risk-of-bias studies, outliers), random effects analysis

#	Atlas label	hem	*x*	*y*	*z*	size	*n*	τ^2	*r*	SEM	*z-score*	p_{FWER}
1	Ant. cingulate g (48%), paracingulate g (28%)	L	-6	6	40	48	373	0.01	-0.27	0.06	4.49	.028
2	SMA (63%), superior frontal g (9%)	R	6	4	58	34	373	0.00	-0.25	0.05	4.66	.018
3	Precentral g (11%), post. cingulate g (10%)	R	16	-18	40	15	372	0.02	-0.26	0.07	3.99	.036
4	Thalamus (98%), prefrontal- (48%†) / premotor- (26%†) subportion	R	18	-18	10	14	372	0.00	-0.25	0.05	4.62	.025
5	Thalamus (99%), prefrontal- (59%†) / temporal- (39%†) subportion	L	-10	-8	12	10	373	0.00	-0.26	0.06	4.83	.018
6	Superior frontal g (8%), SMA (7%)	R	12	6	60	8	373	0.00	-0.25	0.05	4.71	.017
7	Thalamus (7%), prefrontal- (24%†) / post.-parietal- (6%†) subportion	R	18	-6	16	5	372	0.00	-0.25	0.05	4.69	.023
8	Central operculum (48%), insula (17.5%)	R	38	-16	18	2	372	0.00	-0.24	0.05	4.52	.040
9	Parietal operculum (30%), ant. supramarginal g (20%)	R	54	-28	28	2	373	0.00	-0.24	0.05	4.54	.047

Significant clusters of correlation between brain activity ($pain_{placebo}$ – $pain_{control}$) and placebo analgesia ($pain_{control}$ – $pain_{placebo}$) at a threshold of $p_{FWER} < .05$, corrected for multiple comparisons. Cluster labels are provided with probability estimates from the Harvard (Sub-)Cortical (unmarked), Thalamic Connectivity (†), or Talairach (*) atlas. [Square brackets] denote comments. “Size” denotes cluster size in voxels of 2*2*2 mm, all other parameters refer to the peak voxel. All voxels listed showed decreased brain activity with increasing behavioral placebo analgesia, no voxel with positive correlations reached the threshold of statistical significance after correcting for multiple comparisons. Source data (results as 3d-volumes) are provided at https://osf.io/n9mb3/.

Abbreviations: Ant, anterior; B, bilateral; g, gyrus; hem, hemisphere; L, left; perm, permutation test; PL, posterior lobe; post, posterior; R, right; sup, superior; TOFC, Temporal Occipital Fusiform Cortex; WM, white matter.

Supplementary Table 9A: clusters of significant increase in pain-related activity — full sample, random effects analysis

#	Atlas label	hem	x	y	z	size	*n*	τ^2	*g*	SEM	*z*-score	p_{FWER}
1	Frontal pole (10%), insula (6%), [large cluster spanning insula, DLPFC, SII]	R	36	8	8	11834	603	0.45	1.68	0.18	9.33	.000
2	Insula (8%), frontal pole (8%), [large cluster spanning insula, DLPFC, SII]	L	-32	18	4	8808	603	0.50	1.52	0.19	8.15	.000
3	Paracingulate g (23.4%), ant. cingulate g (19%)	R	2	18	46	3449	603	0.37	1.18	0.16	7.28	.000
4	Cerebellum, crus I (39%º), lobule VI (15%º)	L	-30	-66	-30	1219	598	0.42	0.87	0.17	5.28	.000
5	Post. cingulate g (32%)	R	2	-28	26	560	603	0.51	1.03	0.18	5.67	.000
6	Caudate (26%)	R	14	10	0	338	603	0.33	0.92	0.15	6.07	.000
7	Cerebellum, crus I (63%º), lobule VI (11%º)	R	28	-66	-32	288	597	0.37	0.76	0.16	4.83	.000
8	Thalamus, prefrontal (14%†) / premotor (3%†) subportion	R	12	-12	2	128	603	0.55	0.73	0.19	3.92	.045
9	Precuneus (42%), cuneus (13%)	R	12	-70	38	105	603	0.25	0.69	0.13	5.25	.000
10	Cerebellum, crus II (77%º), crus I (6%º)	R	10	-84	-30	10	586	0.19	0.53	0.12	4.30	.029
11	Cerebellum, lobules I-VI (97%º)	L	-4	-50	-10	6	603	0.18	0.51	0.12	4.32	.028
12	Precuneus (40%), cuneus (8%)	L	-10	-72	38	6	603	0.37	0.62	0.15	4.04	.031

Significant clusters of activation and de-activation for the contrast pain – baseline (pooled across placebo and control conditions) at a threshold of p_{FWER} < .05, corrected for multiple comparisons. Cluster labels are provided with probability estimates from the Harvard (Sub-)Cortical (unmarked), Thalamic Connectivity (†), Probabilistic Cerebellarº) or Talairach (*) atlas. [Square brackets] denote comments. "Size" denotes cluster size in voxels of 2*2*2 mm, all other parameters refer to the peak voxel. Source data (results as 3d-volumes) are provided at https://osf.io/n9mb3/.

Abbreviations: Ant, anterior; AL, anterior lobe; C, cortex; DLPFC, dorso-lateral prefrontal cortex; g, gyrus; inf, inferior; perm, permutation test; post, posterior; PL, posterior lobe; SII, secondary somatosensory cortex; sup., superior.

Supplementary Table 9B: clusters of significant decrease in pain-related activity — full sample, random effects analysis

#	Atlas label	hem	*x*	*y*	*z*	size	*n*	τ^2	*g*	SEM	*z*-score	p_{FWER}
1	Paracingulate g (27%), frontal medial c (24%)	B	-2	48	-14	1131	590	0.16	-0.63	0.12	-5.48	.000
2	Precuneus (45%), post. cingulate g (29%)	B	2	-58	22	1118	601	0.14	-0.74	0.11	-6.63	.000
3	Sup. lateral occipital c (54%), angular g (7%)	R	46	-70	26	524	603	0.24	-0.79	0.13	-5.97	.000
4	Sup. lateral occipital c (61%), angular g (4%)	L	-36	-80	28	465	603	0.42	-0.82	0.16	-4.97	.000
5	Post. temporal fusiform c (38%), post. parahippocampal g (29%)	L	-30	-38	-18	193	603	0.28	-0.73	0.14	-5.25	.000
6	Post. temporal fusiform c (40%), post. parahippocampal g (25%)	R	30	-36	-20	161	602	0.46	-0.79	0.17	-4.65	.001
7	Occipital pole (32%), inf. lateral occipital c (26%)	R	28	-94	-6	77	601	0.47	-0.64	0.17	-3.77	.041
8	Postcentral g (49%), precentral g (8%)	L	-46	-22	60	69	586	0.28	-0.68	0.14	-4.82	.004
9	Occipital pole (27%), inf. lateral occipital c (26%)	L	-30	-92	-8	65	603	0.36	-0.59	0.15	-3.89	.041
10	Middle (28%) / superior frontal g (24%)	R	24	28	42	64	602	0.12	-0.50	0.10	-4.89	.000
11	Postcentral (40%) g, precentral (37%) g	L	-60	-6	32	25	601	0.12	-0.48	0.10	-4.73	.004
12	Ant. (15%) / post. middle temporal (39%) g	L	-58	-6	-20	18	598	0.21	-0.52	0.12	-4.20	.018
13	Superior (29%) / middle frontal (22%) g	L	-22	26	44	15	603	0.10	-0.43	0.10	-4.46	.027

Significant clusters of activation and de-activation for the contrast pain – baseline (pooled across placebo and control conditions) at a threshold of $p_{FWER} < .05$, corrected for multiple comparisons. Cluster labels are provided with probability estimates from the Harvard (Sub-)Cortical (unmarked), Thalamic Connectivity (†), Probabilistic Cerebellarº) or Talairach (*) atlas. [Square brackets] denote comments. "Size" denotes cluster size in voxels of 2*2*2 mm, all other parameters refer to the peak voxel. Source data (results as 3d-volumes) are provided at https://osf.io/n9mb3/.
Abbreviations: Ant, anterior; AL, anterior lobe; C, cortex; DLPFC, dorso-lateral prefrontal cortex; g, gyrus; inf, inferior; perm, permutation test; post, posterior; PL, posterior lobe; SII, secondary somatosensory cortex; sup., superior.

Supplementary Table 10: clusters of placebo-treatment induced reduction in pain-related activity — full sample, random effects analysis

#	Atlas label	hem	*x*	*y*	*z*	size	*n*	τ^2	*g*	SEM	*z*-score	p_{FWER}
1	Insula (64.5%)	R	38	8	0	2	603	0.00	-0.17	0.04	-4.16	.040
2	Corpus callosum (100%*) [near splenium]	L	-6	-32	12	2	602	0.00	-0.19	0.05	-3.88	.034
3	Cerebellum, crus I (83%º)	L	-40	-64	-24	1	594	0.00	-0.17	0.04	-3.92	.049

Significant peak voxel of activation and de-activation for the contrast $pain_{placebo}$ – $pain_{control}$ at a threshold of $p_{FWER} < .05$, corrected for multiple comparisons. "Size" refers to the number of contiguous voxels (2*2*2 mm) surpassing voxel-level significance, all other parameters refer to the peak voxel. Labels are provided with probability estimates from the Harvard (Sub-)Cortical if not denoted otherwise, or using the Cerebellar (º), or Talairach (*) atlas. No voxel showing positive activation changes reached the significance threshold. Source data (results as 3d-volumes) are provided at https://osf.io/n9mb3/.

Abbreviations: hem, hemisphere; L, left; R, right; p_{FWER}, permutation-based *p*-value corrected for multiple comparisons using the *z*-max method (family-wise error level).

Supplementary Table 11A: clusters of placebo-treatment induced increase in pain-related activity — full sample, fixed effects analysis

#	Atlas label	hem	*x*	*y*	*z*	size	*n*	τ^2	*g*	SEM	*z*-score	p_{FWER}
1	Sup. lateral occipital c (71%) [bordering parietal cortex]	L	-34	-80	42	10	565	0.03	0.19	0.03	5.80	.003
2	Middle frontal g (58%), frontal pole (11%)	R	46	32	36	6	572	0.04	0.22	0.04	5.92	.002
3	Precuneus (35%), sup. lateral occipital c (9 %)	L	-8	-68	50	5	603	0.05	0.17	0.04	4.14	.019
4	Frontal pole (34%)	R	28	52	-4	3	569	0.04	0.18	0.04	4.25	.017
5	Angular g (7%), sup. parietal lobule (6%)	R	30	-50	36	3	603	0.02	0.18	0.04	4.23	.017
6	Post. middle temporal g (61%), post. inferior temporal g (6%)	R	64	-20	-18	2	601	0.02	0.19	0.04	4.58	.011
7	Middle frontal g (31%), inferior frontal g, pars opercularis (13%)	L	-42	10	32	2	603	0.05	0.16	0.04	4.29	.033
8	Angular g (35%), sup. parietal lobule (17%)	R	40	-54	42	2	603	0.02	0.15	0.04	4.18	.026
9	Amygdala (8%)	L	-32	-8	-16	1	603	0.11	0.20	0.04	5.08	.008
10	Frontal pole (93%)	L	-44	48	4	1	582	0.02	0.15	0.04	4.11	.034
11	Angular gyrus (37%), sup. lateral occipital c (6%)	R	44	-54	42	1	603	0.02	0.16	0.04	3.90	.046
12	Angular gyrus (34%), sup. lateral occipital c (26%)	R	46	-56	52	1	603	0.02	0.16	0.04	3.97	.042

Significant clusters of activation and de-activation for the contrast $pain_{placebol}$ – $pain_{control}$ at a threshold of $p_{FWER} < .05$, corrected for multiple comparisons. Note that fixed effects analysis does not account for between study differences in effect sizes. Cluster labels are provided with probability estimates from the Harvard (Sub-)Cortical (unmarked), Probabilistic Cerebellar (º) or Talairach (*) atlas. [Square brackets] denote comments. "Size" denotes cluster size in voxels of 2*2*2 mm, all other parameters refer to the peak voxel. Source data (results as 3d-volumes) are provided at https://osf.io/n9mb3/.
Abbreviations: g, gyrus; hem, hemisphere; L, left; perm, permutation test; PL, posterior lobe; post, posterior; R, right; sup, superior; TOFC, Temporal Occipital Fusiform Cortex; WM, white matter.

Supplementary Table 11B: clusters of placebo-treatment induced reductions in pain-related activity — full sample, fixed effects analysis

#	Atlas label	hem	*x*	*y*	*z*	size	*n*	τ^2	*g*	SEM	*z*-score	p_{FWER}
1	Putamen (77%)	L	-24	2	-6	36	598	0.06	-0.22	0.05	-4.98	.003
2	Insula (53%)	R	36	8	0	8	603	0.00	-0.17	0.04	-4.22	.018
3	Parietal white matter (100%*)	L	-26	-52	30	2	580	0.04	-0.19	0.03	-5.37	.009
4	Cerebellum, crus I (85%º)	L	-44	-62	-26	1	586	0.00	-0.16	0.04	-3.95	.045
5	Cerebellum, crus I (83%º)	L	-40	-64	-24	1	594	0.00	-0.17	0.04	-3.92	.046
6	Corpus callosum (100%*) [near splenium]	L	-2	-36	6	1	592	0.00	-0.18	0.05	-3.88	.049
7	Corpus callosum (100%*) [near splenium]	L	-6	-32	12	1	602	0.00	-0.19	0.05	-3.88	.045

Significant clusters of activation and de-activation for the contrast $pain_{placebo}$ – $pain_{control}$ at a threshold of $p_{FWER} < .05$, corrected for multiple comparisons. Note that fixed effects analysis does not account for between study differences in effect sizes. Cluster labels are provided with probability estimates from the Harvard (Sub-)Cortical (unmarked), Probabilistic Cerebellar (º) or Talairach (*) atlas. [Square brackets] denote comments. "Size" denotes cluster size in voxels of 2*2*2 mm, all other parameters refer to the peak voxel. Source data (results as 3d-volumes) are provided at https://osf.io/n9mb3/.
Abbreviations: g, gyrus; hem, hemisphere; L, left; perm, permutation test; PL, posterior lobe; post, posterior; R, right; sup, superior; TOFC, Temporal Occipital Fusiform Cortex; WM, white matter.

Supplementary Table 12: clusters showing a significant negative correlation between brain activity and behavioral placebo analgesia — full sample (sans between-subject studies), random effects analysis

#	Atlas label	hem	*x*	*y*	*z*	size	*n*	τ^2	*r*	SEM	*z*-score	p_{FWER}
1	Thalamus (99%), prefrontal- (63%†) / premotor- (18%†) subportion	R	10	-18	6	46	460	0.01	-0.26	0.05	4.89	.010
2	Thalamus (97%), prefrontal- (65%†) / temporal- (34%†) subportion	L	-10	-8	12	19	460	0.00	-0.24	0.05	5.05	.005
3	Ant. cingulate g (41%), paracingulate g (27%)	L	-4	8	40	19	460	0.01	-0.23	0.05	4.48	.039
4	Inferior frontal g, pars triangularis (10%)	R	54	20	-6	1	413	0.00	-0.23	0.05	4.35	.049
5	Precentral g (15%), post. cingulate g (12%)	R	16	-20	40	1	437	0.01	-0.24	0.05	4.55	.045
6	SMA (63%), superior frontal g (15%)	R	4	6	64	1	460	0.00	-0.23	0.05	4.57	.043

Significant clusters of correlation between brain activity ($pain_{placebo} – pain_{control}$) and placebo analgesia ($pain_{control} — pain_{placebo}$) at a threshold of $p_{FWER} < .05$, corrected for multiple comparisons. Cluster labels are provided with probability estimates from the Harvard (Sub-)Cortical (unmarked), Thalamic Connectivity (†), or Talairach (*) atlas. [Square brackets] denote comments. “Size” denotes cluster size in voxels of 2*2*2 mm, all other parameters refer to the peak voxel. All voxels listed showed decreased brain activity with increasing behavioral placebo analgesia, no voxel with positive correlations reached the threshold of statistical significance after correcting for multiple comparisons. Source data (results as 3d-volumes) are provided at https://osf.io/n9mb3/.
Abbreviations: Ant, anterior; B, bilateral; g, gyrus; hem, hemisphere; L, left; perm, permutation test; PL, posterior lobe; post, posterior; R, right; sup, superior; TOFC, Temporal Occipital Fusiform Cortex; WM, white matter.

Supplementary References

1. Zunhammer, M., Bingel, U. & Wager, T. D. Placebo Effects on the Neurologic Pain Signature: A Meta-analysis of Individual Participant Functional Magnetic Resonance Imaging Data. *JAMA Neurol.* (2018). doi:10.1001/jamaneurol.2018.2017
2. Atlas, L. Y. & Wager, T. D. A meta-analysis of brain mechanisms of placebo analgesia: consistent findings and unanswered questions. *Handb. Exp. Pharmacol.* **225**, 37–69 (2014).
3. Wager, T. D. & Atlas, L. Y. The neuroscience of placebo effects: Connecting context, learning and health. *Nat. Rev. Neurosci.* **16**, 403–418 (2015).
4. Fehse, K. & Maikowski, L. Placebo responses to original vs generic ASA brands during exposure to noxious heat: a pilot fMRI study of neurofunctional correlates. *Pain Med.* **16**, 1967–1974 (2015).
5. van der Meulen, M., Kamping, S. & Anton, F. The role of cognitive reappraisal in placebo analgesia: An fMRI study. *Soc. Cogn. Affect. Neurosci.* **12**, 1128–1137 (2017).
6. Schenk, L. A., Sprenger, C., Onat, S., Colloca, L. & Büchel, C. Suppression of Striatal Prediction Errors by the Prefrontal Cortex in Placebo Hypoalgesia. *J. Neurosci.* **37**, 9715–9723 (2017).
7. Gollub, R. L. *et al.* A Functional Neuroimaging Study of Expectancy Effects on Pain Response in Patients With Knee Osteoarthritis. *J. Pain* (2018). doi:10.1016/j.jpain.2017.12.260
8. Linnman, C. *et al.* Molecular and functional PET-fMRI measures of placebo analgesia in episodic migraine: Preliminary findings. *NeuroImage Clin.* **17**, 680–690 (2018).
9. Yue, Y. & Collaku, A. Correlation of Pain Reduction with fMRI BOLD Response in Osteoarthritis Patients Treated with Paracetamol: Randomized, Double-Blind, Crossover Clinical Efficacy Study. *Pain Med.* 355–367 (2017). doi:10.1093/pm/pnx157
10. *Cochrane Handbook for Systematic Reviews of Interventions Version 5.1.0 [updated March 2011]*. (The Cochrane Collaboration, 2011).
11. Paludan-Müller, A., Teindl Laursen, D. R. & Hróbjartsson, A. Mechanisms and direction of allocation bias in randomised clinical trials. *BMC Med. Res. Methodol.* **16**, 133 (2016).
12. Hróbjartsson, A., Kaptchuk, T. J. & Miller, F. G. Placebo effect studies are susceptible to response bias and to other types of biases. *J. Clin. Epidemiol.* **64**, 1223–9 (2011).
13. Benedetti, F. Placebo and the new physiology of the doctor-patient relationship. *Physiol. Rev.* **93**, 1207–46 (2013).
14. Kessner, S., Wiech, K., Forkmann, K., Ploner, M. & Bingel, U. The effect of treatment history on

therapeutic outcome: an experimental approach. *JAMA Intern. Med.* **173**, 1468–9 (2013).

15. Gelman, A. & Loken, E. The garden of forking paths: Why multiple comparisons can be a problem, even when there is no “fishing expedition” or “p-hacking” and the research hypothesis was posited ahead of time. *Psychol. Bull.* **140**, 1272–1280 (2014).

16. Kong, J. *et al.* Expectancy and treatment interactions: a dissociation between acupuncture analgesia and expectancy evoked placebo analgesia. *Neuroimage* **45**, 940–9 (2009).

17. Kong, J. *et al.* Brain activity associated with expectancy-enhanced placebo analgesia as measured by functional magnetic resonance imaging. *J. Neurosci.* **26**, 381–8 (2006).

18. Zeidan, F. *et al.* Mindfulness Meditation-Based Pain Relief Employs Different Neural Mechanisms Than Placebo and Sham Mindfulness Meditation-Induced Analgesia. *J. Neurosci.* **35**, 15307–15325 (2015).

19. Schmucker, C. *et al.* Extent of non-publication in cohorts of studies approved by research ethics committees or included in trial registries. *PLoS One* **9**, 1–25 (2014).

20. Wager, T. D. *et al.* Placebo-induced changes in FMRI in the anticipation and experience of pain. *Science* **303**, 1162–7 (2004).

21. Choi, J. C. *et al.* Placebo effects on analgesia related to testosterone and premotor activation. *Neuroreport* **22**, 419–23 (2011).

22. Bingel, U., Lorenz, J., Schoell, E., Weiller, C. & Büchel, C. Mechanisms of placebo analgesia: rACC recruitment of a subcortical antinociceptive network. *Pain* **120**, 8–15 (2006).

23. Huber, A., Lui, F. & Porro, C. A. Hypnotic susceptibility modulates brain activity related to experimental placebo analgesia. *Pain* **154**, 1509–1518 (2013).

24. Freeman, S. *et al.* Distinct neural representations of placebo and nocebo effects. *Neuroimage* **112**, 197–207 (2015).

25. Bingel, U. *et al.* The effect of treatment expectation on drug efficacy: imaging the analgesic benefit of the opioid remifentanil. *Sci. Transl. Med.* **3**, 70ra14 (2011).

26. Rütgen, M. *et al.* Placebo analgesia and its opioidergic regulation suggest that empathy for pain is grounded in self pain. *Proc. Natl. Acad. Sci. U. S. A.* **112**, E5638-46 (2015).

27. Spisák, T. *et al.* Probabilistic TFCE: A generalized combination of cluster size and voxel intensity to increase statistical power. *Neuroimage* **185**, 12–26 (2019).

28. Deeks, J. J. & Higgins, J. P. *Statistical algorithms in Review Manager 5 on behalf of the Statistical Methods Group of The Cochrane Collaboration*. *Documentation* **2010**, (2010).

29. Atlas, L. Y. *et al.* Dissociable influences of opiates and expectations on pain. *J. Neurosci.* **32**, 8053–64

(2012).

30. Friston, K. J., Rotshtein, P., Geng, J. J., Sterzer, P. & Henson, R. N. A critique of functional localisers. *Neuroimage* **30**, 1077–1087 (2006).

31. Lakens, D. Calculating and reporting effect sizes to facilitate cumulative science: a practical primer for t-tests and ANOVAs. *Front. Psychol.* **4**, 1–12 (2013).

32. Borenstein, M., Hedges, L. V, Higgins, J. P. T. & Rothstein, H. R. Effect Sizes Based on Means. in *Introduction to Meta-Analysis* (2009). doi:10.1002/9780470743386.ch6

33. Nichols, T. E. & Holmes, A. P. Nonparametric permutation tests for functional neuroimaging: a primer with examples. *Hum. Brain Mapp.* **15**, 1–25 (2002).

34. Desikan, R. S. *et al.* An automated labeling system for subdividing the human cerebral cortex on MRI scans into gyral based regions of interest. *Neuroimage* **31**, 968–80 (2006).

35. Behrens, T. E. J. *et al.* Non-invasive mapping of connections between human thalamus and cortex using diffusion imaging. *Nat. Neurosci.* **6**, 750–7 (2003).

36. Diedrichsen, J., Balsters, J. H., Flavell, J., Cussans, E. & Ramnani, N. A probabilistic MR atlas of the human cerebellum. *Neuroimage* **46**, 39–46 (2009).

37. Lancaster, J. L. *et al.* Bias between MNI and talairach coordinates analyzed using the ICBM-152 brain template. *Hum. Brain Mapp.* **28**, 1194–1205 (2007).

38. MacCallum, R. C., Zhang, S., Preacher, K. J. & Rucker, D. D. On the practice of dichotomization of quantitative variables. *Psychol. Methods* **7**, 19–40 (2002).

39. Yeo, B. T. T. *et al.* The organization of the human cerebral cortex estimated by intrinsic functional connectivity. *J. Neurophysiol.* **106**, 1125–1165 (2011).

40. Faillenot, I., Heckemann, R. A., Frot, M. & Hammers, A. Macroanatomy and 3D probabilistic atlas of the human insula. *Neuroimage* **150**, 88–98 (2017).

41. Krauth, A. *et al.* A mean three-dimensional atlas of the human thalamus: Generation from multiple histological data. *Neuroimage* **49**, 2053–2062 (2010).
