# Supplementary material for: Meta-analysis of neural systems underlying placebo analgesia from individual participant fMRI data
Source: Nat Commun. 2021 Mar 2;12:1391. doi: 10.1038/s41467-021-21179-3 (PMC7925520; doi:10.1038/s41467-021-21179-3)
Supplement: Supplementary file 3 — Reporting Summary [file 41467_2021_21179_MOESM3_ESM.pdf]

nature research

Corresponding author(s): Tor Wager, Ulrike Bingel

Last updated by author(s): Sep 23, 2020

Reporting Summary

Nature Research wishes to improve the reproducibility of the work that we publish. This form provides structure for consistency and transparency in reporting. For further information on Nature Research policies, see our Editorial Policies and the Editorial Policy Checklist.

Statistics

For all statistical analyses, confirm that the following items are present in the figure legend, table legend, main text, or Methods section.

n/a	Confirmed	
☐	☒	The exact sample size (*n*) for each experimental group/condition, given as a discrete number and unit of measurement
☐	☒	A statement on whether measurements were taken from distinct samples or whether the same sample was measured repeatedly
☐	☒	The statistical test(s) used AND whether they are one- or two-sided<br>*Only common tests should be described solely by name; describe more complex techniques in the Methods section.*
☐	☒	A description of all covariates tested
☐	☒	A description of any assumptions or corrections, such as tests of normality and adjustment for multiple comparisons
☐	☒	A full description of the statistical parameters including central tendency (e.g. means) or other basic estimates (e.g. regression coefficient) AND variation (e.g. standard deviation) or associated estimates of uncertainty (e.g. confidence intervals)
☐	☒	For null hypothesis testing, the test statistic (e.g. *F*, *t*, *r*) with confidence intervals, effect sizes, degrees of freedom and *P* value noted<br>*Give P values as exact values whenever suitable.*
☒	☐	For Bayesian analysis, information on the choice of priors and Markov chain Monte Carlo settings
☐	☒	For hierarchical and complex designs, identification of the appropriate level for tests and full reporting of outcomes
☐	☒	Estimates of effect sizes (e.g. Cohen's *d*, Pearson's *r*), indicating how they were calculated

Our web collection on statistics for biologists contains articles on many of the points above.

Software and code

Policy information about availability of computer code

Data collection	No specific software was used for data collection.
Data analysis	MATLAB v2016b with toolboxes SPM v12, CANlab and custom code available at www.github.com/mzunhammer/PlaceboMetaAnalysis. In addition: fsl v5.0.10 (automated cluster labeling), MRIcroGL v28.5.2017 (visualization).

For manuscripts utilizing custom algorithms or software that are central to the research but not yet described in published literature, software must be made available to editors and reviewers. We strongly encourage code deposition in a community repository (e.g. GitHub). See the Nature Research guidelines for submitting code & software for further information.

Data

Policy information about availability of data

All manuscripts must include a data availability statement. This statement should provide the following information, where applicable:

- Accession codes, unique identifiers, or web links for publicly available datasets
- A list of figures that have associated raw data
- A description of any restrictions on data availability

Results as 3d-volumes are provided at https://osf.io/n9mb3/. Participant-level source data are available from the authors upon reasonable request and with permission of the Placebo Imaging Consortium.

Field-specific reporting

Please select the one below that is the best fit for your research. If you are not sure, read the appropriate sections before making your selection.

☒ Life sciences ☐ Behavioural & social sciences ☐ Ecological, evolutionary & environmental sciences

For a reference copy of the document with all sections, see nature.com/documents/nr-reporting-summary-flat.pdf

Life sciences study design

All studies must disclose on these points even when the disclosure is negative.

Sample size	603 individual participants within 20. Since this is a meta-analysis of individual participant data the sample size was not determined a-priori but based on the available/shared data.
Data exclusions	In literature screening 68 out of 96 full texts screened were excluded from analysis based on pre-established eligibility criteria (see: Supplementary Methods and Results, Supplementary Figure S1, Table S1). In analysis, brain-voxels missing in > 10% of participants (total sample) were excluded from further analysis (see: Supplementary Methods and Results) to keep the sample-size comparable across the brain. This exclusion criterion was not pre-established, but employed post-hoc. No other data-exclusion was performed in main analysis.
Replication	A "conservative analysis" was performed excluding suspected single-subject outliers and high risk-of-bias studies. Similar results were obtained. In addition, a fixed-effects analysis was performed to highlight the influence of between-study heterogeneity. (see: Supplementary Appendix)
Randomization	NA to a Meta-Analysis. For Group definitions see: eTables 3
Blinding	Meta-analysts were not blinded to the group (placebo vs control) labels as it was deemed difficult/futile. Summary results for all included studies were already published. The analysts involved were intimately familiar with the results of these published studies. In many cases one look at study-level summary images would have unblinded analysts.

Reporting for specific materials, systems and methods

We require information from authors about some types of materials, experimental systems and methods used in many studies. Here, indicate whether each material, system or method listed is relevant to your study. If you are not sure if a list item applies to your research, read the appropriate section before selecting a response.

Materials & experimental systems

n/a	Involved in the study	
☒	☐	Antibodies
☒	☐	Eukaryotic cell lines
☒	☐	Palaeontology and archaeology
☒	☐	Animals and other organisms
☐	☒	Human research participants
☒	☐	Clinical data
☒	☐	Dual use research of concern

Methods

n/a	Involved in the study	
☒	☐	ChIP-seq
☒	☐	Flow cytometry
☐	☒	MRI-based neuroimaging

Human research participants

Policy information about studies involving human research participants

Population characteristics	Healthy volunteers of both sexes, as recruited in 20 original studies included in this participant-level meta-analysis. See: Table 1
Recruitment	Investigators of all eligible studies were contacted and invited to share data (see: Supplementary Methods and Results)
Ethics oversight	The present participant-level meta-analysis was solely based on fully anonymized participant data (normalized statistical summary images at participant level and associated demographic/behavioral data, participant IDs were anonymized). The original studies included were all approved separately by local ethics committees (as guaranteed by the members of the Placebo Imaging Consortium). The meta-analysis itself was exempt from ethics approval.

Note that full information on the approval of the study protocol must also be provided in the manuscript.

Magnetic resonance imaging

Experimental design

Design type	Participant level meta-analysis based on functional neuroimaging experiments, mix of block & event-related designs.
Design specifications	See: Methods, Table 1 and eTables 2-7
Behavioral performance measures	See: eTable 2

Acquisition

Imaging type(s)	19x fMRI 1xASL
Field strength	See: eTable 4, 4x 1.5T, 16x3T
Sequence & imaging parameters	Various, See: eTable 4
Area of acquisition	Whole Brain

Diffusion MRI ☐ Used ☒ Not used

Preprocessing

Preprocessing software	Various, see: eTable 5
Normalization	Various, see: eTable 5
Normalization template	Various, see: eTable 5, the MNI152 brain-template, as implemented in SPM12, was used for meta-analysis
Noise and artifact removal	Various, see: eTable 5
Volume censoring	Various, see: eTable 5

Statistical modeling & inference

Model type and settings	Meta-analysis: Hedge`s g summarized using the Generic Inverse Variance Method in combination with a pseudo-z-based permutation test.
Effect(s) tested	Pain vs Baseline, Placebo vs Control, Correlation of Brain Activity vs Pain Rating for the contrast of Placebo vs Control

Specify type of analysis: ☐ Whole brain ☐ ROI-based ☒ Both

Anatomical location(s)	Whole brain: The fsl (version 5.0.10) function "cluster", as implemented in the atlasquery automation script (autoaq), was used to label thresholded summary images, automatically (s. Supplement p.9).<br>ROIs: (i) canonical large-scale functional connectivity networks, ref32 (resting-state), as well as (ii) insular sub-regions (anatomy based) ref33, and (iii) thalamic nuclei (anatomy based) ref34 (s. Manuscript, p.8)
Statistic type for inference (See Eklund et al. 2016)	Voxel-level: FWE-corrected for multiple comparisons (maximum-z method: permutation-based w tail fitting) alpha level of p < .05, also p < .01 uncorrected for visualization.<br>Cluster level: FWE-corrected for multiple comparisons (probabilistic threshold-free cluster enhancement) alpha level of p < .05, also p < .01 uncorrected for visualization.<br>ROI level: p < .05, uncorrected for multiple comparisons
Correction	See: above.

Models & analysis

n/a	Involved in the study
☒	☐ Functional and/or effective connectivity
☒	☐ Graph analysis
☒	☐ Multivariate modeling or predictive analysis
